# Gut dysbiosis impacts estrogen levels in APP/PS1 transgenic female mice

**DOI:** 10.1080/19490976.2025.2599525

**Published:** 2025-12-18

**Authors:** Ivonne Sagrario Romero-Flores, Jaime García-Mena, Claudia Perez-Cruz

**Affiliations:** a Genetics and Molecular Biology Department, Center for Research and Advanced Studies of the National Polytechnic Institute (Cinvestav), Laboratory of Reference and Support For the Characterization of Genomes, Transcriptomes and Microbiomes, Mexico City, Mexico; b Pharmacology Department, Center for Research and Advanced Studies of the National Polytechnic Institute (Cinvestav), Laboratory of Neuroplasticity and Neurodegeneration, Mexico City, Mexico

**Keywords:** Beta-glucuronidase, gut dysbiosis, estrobolome, Alzheimer's disease, estrogen

## Abstract

Alzheimer's disease (AD) is the most common form of dementia, with a higher prevalence in women than in men. It has been suggested that the decline in estrogen production after menopause may increase the risk of developing dementia. Additionally, patients with AD often display dysbiosis of the gut microbiota (GM), even in the early stages of the disease. The GM plays a crucial role in modulating systemic estrogen levels through a mechanism known as the *estrobolome*. However, it remains unclear whether gut dysbiosis contributes to estrogen imbalance and subsequent cognitive decline in women. In this study, we aim to investigate whether alterations in the GM impact estrogen availability and cognitive function in 6-month-old female APP/PS1 (TG) mice compared to age-matched wild-type (WT) littermates. We included a group of both WT and TG mice treated with a broad-spectrum antibiotic cocktail (ABX) for one month to modify their GM composition. Our results revealed that TG mice exhibited a dysfunctional *estrobolome* characterized by a decreased abundance of *Limosilactobacillus* and *Lactobacillus*, an increased abundance of *Ligilactobacillus*, and reduced activity of the β-glucuronidase enzyme in fecal samples. Additionally, TG female mice showed low bioavailability of estradiol, disrupted estrous cycle, and cognitive impairments. Notably, WT-ABX mice displayed gut dysbiosis, marked by a decrease in the relative abundances of *Limosilactobacillus* and *Lactobacillus*, as well as reduced β-glucuronidase activity. Moreover, WT-ABX exhibited altered estradiol levels and cognitive impairments compared to WT controls. Therefore, our findings suggest that gut dysbiosis may be a contributing factor to female vulnerability in developing dementia by disrupting hormonal levels and cognitive function.

## GRAPHICAL ABSTRACT

**Figure:**
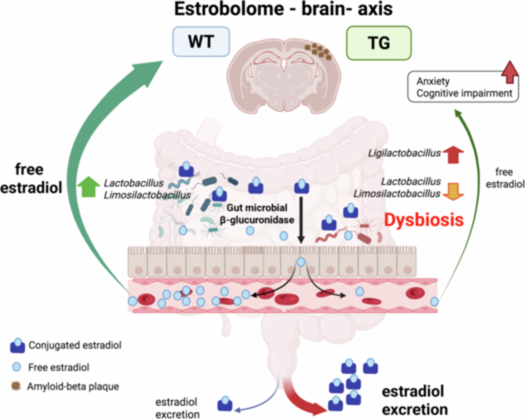

## Introduction

Alzheimer's disease (AD) is characterized by behavioral symptoms such as memory loss, cognitive decline, and difficulties in emotional regulation.^1^ The primary risk factor for AD is advanced age, followed by genetic, environmental, and modifiable lifestyle factors.^2^ Recent data indicate that nearly two-thirds of AD patients are women, with a faster progression of the disease compared to men.^3^^,^^4^ The biological mechanisms contributing to the increased vulnerability of women to AD remain largely unclear. However, it has been suggested that sex steroid hormones, particularly 17-β estradiol (estradiol), may play a crucial role in the development of AD.^5^ Estradiol is a potent neuroprotective hormone,^6^ as its depletion following oophorectomy is associated with a 70% increased risk of developing AD.^7^^,^^8^ Additionally, ovariectomy results in cognitive impairments,^9^^,^^10^ an earlier onset, and greater amyloid-β deposition in transgenic AD mice.^11^

Estrogens are produced in the ovaries and are released into the bloodstream to reach target organs, including the brain. Thereafter, estrogen undergoes first-pass metabolism in the liver through methylation, glucuronidation, or sulfonation to enable the excretion of estrogens via bile, urine, and feces. In this study, we focused on glucuronidation. Glucuronidation requires UDP-glucuronyl-transferase to form estrogen-glucuronide, which is then excreted in the feces. In the distal gut, certain bacteria produce β-glucuronidases that catalyze the removal of glucuronic acid, resulting in the deconjugation of estrogen.^12^ This free active form of estrogen is then reintroduced into the enterohepatic circulation.^13^^,^^14^ Therefore, gut bacteria modulate estrogen levels in the circulation. Middle-aged women with high hormonal levels exhibit greater gut microbiota diversity than low hormone group,^15^ while the enzymatic activity of β-glucuronidase is linked to higher plasma estrogen levels.^16^ The group of gut bacteria involved in estrogen metabolism is collectively known as the “*estrobolome”.*^17^ Thus, the diversity of the gut microbiota regulates the function of the *estrobolome* and, consequently, it is associated with circulating estrogen levels.

Recent studies have shown that AD patients exhibit lower gut microbiota diversity compared to healthy subjects of the same age.^18^^,^^19^ The imbalanced gut microbiota, also known as dysbiosis, worsens as AD progresses.^20^ Dysbiosis has also been observed in mice that overexpress APP and PS1 humanized genes.^21^^,^^22^ However, the relationships among gut dysbiosis, circulating estrogen levels, and cognitive function in females are still not well understood. In this study, we aimed to investigate whether gut microbiota dysbiosis affects circulating estrogen levels, ultimately leading to a negative impact on cognition. We utilized 6-month-old female APP/PS1 (TG) mice and their wild-type (WT) littermates, with or without a broad-spectrum antibiotic treatment, to modify the diversity of the gut microbiota. We evaluated *estrobolome* function by measuring free estradiol and conjugated estradiol-glucuronide levels in plasma, brain, and feces, as well as β-glucuronidase activity in fecal samples. Additionally, we assessed the gut microbiota profile and cognitive functions of all the animals. Our findings suggest that gut dysbiosis in TG female mice disrupts the *estrobolome* function, leading to lower plasma estradiol levels, higher estradiol excretion, and a negative association with the fertility index.

## Materials and methods

### Mice model

Hemizygous female and male mice (B6.Cg-Tg (APPswe, PSEN1dE9)85Dbo/Mmjax, RRID: MMRRC_034832-JAX; APP/PS1) expressing a chimeric mouse/human APP (Mo/HuAPP695swe) and a mutant human PS1 (PS1-dE9) transgene on a C57BL/6 genetic background and their wild-type (WT) littermates were used. The APP/PS1 (TG) mouse strain was obtained from the Mutant Mouse Resource and Research Center (MMRRC) at The Jackson Laboratory, an NIH-funded strain repository, and was donated to the MMRRC by David Borchelt, Ph.D., McKnight Brain Institute, University of Florida, USA.

For genotyping, DNA from a tail sample was isolated using the DNAzol® Reagent-Genomic DNA Isolation Kit (Invitrogen, Thermo Fisher Scientific). APP/PS1 mice were identified by the presence of two bands (PS1: 608 base pair; IL-2: 324 base pair), and wild-type mice were identified by the presence of one band (IL-2: 324 base pair).

### Experiment design

Animal management and health status were supervised by licensed veterinarian care and approved by the Bioethics Committee of the Center for Research and Advanced Studies (Protocol No. 235–16), following the Mexican Official Standard NOM-062-ZOO-1999 and the principles outlined in the 8th edition of the National Institute of Health (NIH) guide for the care and use of laboratory animals and endorsed by the Institute for Laboratory Animal Research, the Division on Earth and Life Studies and the National Research Council of the National Academies.

Mice were maintained under controlled conditions (12/12 h reverse light–dark cycle, 20 °C, and 40%–50% relative humidity) with *ad libitum* access to food (Rodent LabDiet 5058), provided continuously since weaning. The mice were housed in groups of 3–4 animals of the same genotype until 5 months of age. Then, the mice were randomly assigned to two experimental groups: control (vehicle, C) groups included APP/PS1 (TG) [*n* = 7] and wild-type (WT) [*n* = 8] female mice, which received autoclaved water; treated (antibiotic-treated, ABX) groups included APP/PS1 (TG-ABX) [*n* = 7] and wild-type (WT-ABX) [*n* = 7] mice, which received a combination of antibiotics [ampicillin (1 g/L), neomycin (1 g/L), metronidazole (1 g/L), and vancomycin (0.5 g/L)] in their drinking water for 1 month (Supplementary Material Figure S1, Experimental design). The antibiotic cocktail treatment effectively alters the gut microbiota diversity^23^^,^^24^ and results in an enlargement of the cecum, which is indicative of effective microbial suppression, which was confirmed in the ABX-treated mice (WT-ABX and TG-ABX groups; Supplementary Material Figure S2).

We included a group of 6-month-old male APP/PS1 (TG, *n* = 4) and wild-type (WT, *n* = 6) mice to evaluate gender differences in estradiol metabolism and β-glucuronidase activity (Supplementary Material Figure S3).

Single-housed mice were placed in enriched cages (nesting material and objects for exploration) to minimize stress and to allow accurate fecal sample collection and accurate treatment evaluation (ABX). Fresh feces from all the mice were collected prior to sacrifice and stored at −70 °C until analysis.

### Vaginal cytology evaluation

Vaginal cytology was performed daily for 28 d. Initial testing and habituation were performed 3 d prior to the estrous cycle registry. Vaginal smear preparation consisted of inserting a plastic pipette tip filled with 10 µL of phosphate-buffered saline (PBS) solution into the vagina and flushing the solution into the vagina several times until a dense consistency was obtained. The flush containing the dense vaginal fluid was placed on a glass slide and allowed to dry at room temperature for 5 min. The vaginal smears were stained with 400 μL of hematoxylin‒eosin for 5 min, and then the slides were rinsed with running water and viewed at 10× magnification under a bright-field microscope. The stages of the estrous cycle were identified as previously described.^25^^,^^26^

### Cognitive assessment

To control for the influence of hormones on behavior, all cognitive evaluations were performed only during the metestrus/diestrus, the reproductive estrous stages with the lowest estrogen levels.^27^ All behavioral evaluations were performed during the active phase of the animal (dark phase) and after one week of daily handling and habituation to the behavioral room. To evaluate anxiety, we used the elevated plus maze (EPM)^28^ as described in previous studies.^22^ To assess working memory, we used the T-maze test^29^ according to previous studies.^22^ The Novel object recognition (NOR), an appropriate test to evaluate recognition memory that is affected in AD,^30^ was carried out as described in previous studies.^22^ The Morris water maze (WM) was used to evaluate learning and spatial memory.^31^ The WM task was carried out as previously described.^31^^,^^32^ All behavioral tests were recorded and analyzed by autotracking software (ANY-maze, Stoelting Co., Wood Dale, Illinois, USA, Version 7.49).

### Tissue preparation

Once all behavior trials were completed, female mice in the metestrus/diestrus stages were anesthetized with a lethal dose of sodium pentobarbital (150 mg/kg) to obtain circulating blood by cardiac puncture. The blood was centrifuged at 3500 rpm for 15 min at 4 °C to obtain the plasma. The brains were immediately dissected, and both hemispheres were collected. The left hemisphere was immediately placed in a tube, snap-frozen in liquid nitrogen, and subsequently stored at −70 °C until analysis for free and conjugated estradiol levels. The right hemisphere was post-fixed in 4% paraformaldehyde for 72 h at 4 °C for immunohistochemistry (IHC) analysis. For IHC, brain tissue was cryoprotected by immersion in 30% sucrose/water for 3 d. Coronal brain slices (40 μm thickness) were obtained with a sliding microtome (Leica Jung histoslide 2000R). Sections from Bregma −2.80 mm to −2.92 mm^33^ were immersed in cryoprotectant solutions, as previously described.^34^

### Quantification of Aβ plaques in different brain regions

Coronal brain sections were washed overnight in PBS, incubated in 89% formic acid (J.T. Baker) for 15 min and then thoroughly washed with PBS. Then, the slides were boiled in citrate buffer (Sigma) for 10 min and immediately incubated in 0.3% H_2_O_2_ in PBS for 10 min, then washed in 0.2% PBS-Triton and incubated in 0.5% bovine serum albumin (BSA, Sigma) diluted in 0.2% PBS-Triton for 30 min, followed by overnight incubation with an amyloid-β antibody (BAM-10, 1:250 Sigma Cat. No. A3981) diluted in 0.2% PBS-Triton at 4 °C. After being washed in 0.2% PBS-Triton, the sections were incubated with secondary antibody (1:500; Jackson ImmunoRes Cat. No. 115-035-146) in 0.2% PBS-Triton for 2 h at room temperature. Subsequently, the sections were washed with 0.2% PBS-Triton for 10 min, and the antibody binding was visualized and stained with 0.025% 3,3′-diaminobenzidine (DAB Peroxidase Kit; Vector Laboratories) with 0.01% H_2_O_2_ as a catalytic agent. The sections were washed with PBS and mounted on glass slides. sections were processed without the primary antibody. To analyze the amyloid-β (Aβ) aggregates, brightfield images were obtained LEICA ICC50W optical microscope equipped with a digital camera. All images were taken with the same light exposure under a 10× objective. The software ImageJ was used to count the number of Aβ plaques, and the plugin “measure” was used to determine the plaque size. The total number of Aβ plaques was counted in a region of interest (ROI) of the visual, auditory, retrosplenial, and entorhinal cortices and CA1 region of the hippocampus across three separate brain sections per animal per group. Only plaques with an area ≥100 μm^2^ were quantified.

### Determination of free and conjugated-estradiol

Free (17β-estradiol) and conjugated (estradiol 17-β-D-glucuronide) estradiol levels were determined in the plasma (100 μL), brain (30 mg), and fecal samples (30 mg) of each animal. Briefly, each sample was vortex-mixed for 4 min with 1 mL of cold buffer (10 mM Tris [Bio-Rad], 1.5 mM EDTA [Sigma-Aldrich], pH 7.4), followed by centrifugation (12,000 × g, 4 °C, 10 min). The supernatants were collected (up to 1 mL), and 1 mL of deionized water was added before being loaded onto previously activated C18 columns (Bond Elut C18, 3 mL, Agilent Technologies No. 12102028). Once the sample was introduced, a mixture of HPLC-grade methanol (Sigma-Aldrich) in water was used to wash and eluted with HPLC-grade methanol (Sigma-Aldrich). Each eluate was collected in a glass test tube and placed in a water bath at 60 °C until the methanol completely evaporated. Thereafter, 2 mL of HPLC-grade methanol was added to the tubes, followed by sonication until a homogenized sample was obtained and analyzed by high-performance liquid chromatography (HPLC; PerkinElmer Series N3896). The mobile phase consisted of 40% solution A (KH_2_PO_4_, pH unadjusted, J.T. Baker) and 60% solution B (45% acetonitrile, J.T. Baker and 15% methanol, Sigma-Aldrich). The detection threshold for standard curves for free estradiol (17β-estradiol; Sigma-Aldrich No. E8875) was 50–1000 pg/μL, and for conjugated-estradiol (estradiol 17-β-D-glucuronide; Cayman Chemical No. 16156) was 1000–10,000 pg/μL. The amount of free and conjugated-estradiol was expressed in pg/μL plasma or pg/mg for brain or fecal samples.

Total estradiol levels from plasma and fecal samples were analyzed from female and male mice. 100 mg of feces were dried for up to 7 d in a sterile container containing calcium chloride as a desiccant. Subsequently, 3 mL of methanol (J.T. Baker) was added to each sample, followed by overnight shaking at 2200 rpm. The samples were then centrifuged at 3000 rpm for 30 min. The supernatant was collected into a glass test tube and placed in a 60 °C water bath until the methanol was completely evaporated. Next, 2 mL of methanol was added to the dried extract, and the mixture was sonicated until homogenized. Total estradiol levels were measured using enzyme-linked immunosorbent assay ELISA kits (Estradiol: MBS27001152, MyBioSource).

### β-Glucuronidase activity assay

Bacterial β-glucuronidase activity was determined in fecal samples using a colorimetric method. For each animal, a 200 mg fecal sample was used. Briefly, 300 µL of RIPA lysis buffer was added to each sample, and homogenized by mechanical disruption using a tissue grinder for 3 min. The samples were centrifuged at 14,000  rpm for 15 min at 4 °C, after which the supernatant was recovered. The extracted enzyme fraction was used to measure the enzymatic activity according to the enzymatic assay of β-glucuronidase (EC 3.2.1.31) from *Escherichia coli* (Sigma Aldrich). The enzymatic activity was expressed in units/g fecal.

### Liver function and morphology

To assess liver morphology, a portion of a lobe was collected and postfixed in 4% paraformaldehyde for 72 h at 4 °C for paraffin-embedding. Liver tissue sections (8 μm thick) were obtained with a cryostat and dehydrated through absolute alcohol and rehydrated in water. The slides were placed in hematoxylin staining for 15 min, rinsed in water, differentiated in 1% acidic alcohol, rinsed with water, and immersed in ammonia water (NH4OH) until blue coloration was obtained. After being rinsed in water for 10 min, the sections were placed in 80% ethanol for 2 min and counterstained with eosin solution for 2 min. The sections were dehydrated in 96% xylene and mounted in resin. Digital images were captured using a LEICA ICC50W optical microscope equipped with a digital camera, which uses the same brightness and contrast within a plate.

Liver damage was measured by determining the activity of Alanine Aminotransferase (ALT) in plasma samples. A calibration curve was obtained via a colorimetric method.^35^ Test tubes containing increasing concentrations of substrate solution (L-alanine, Sigma-Aldrich; α-ketoglutaric acid disodium salt hydrate, Sigma-Aldrich), and pyruvate solution (sodium pyruvate, Sigma-Aldrich) were prepared. For ALT activity, test tubes containing substrate solution (L-alanine, Sigma-Aldrich; α-ketoglutaric acid disodium salt hydrate, Sigma-Aldrich) and plasma samples were incubated for 60 min at 37 °C. Then, chromogenic reagent (2,4-dinitrofenilhidracina, Sigma-Aldrich) was added, and incubated at 37 °C for 15 min. Finally, 0.4 N NaOH was added, and the absorbance was measured at 515 nm. The results are expressed as µmol of pyruvate/L/min.

### Determination of short-chain fatty acids

Dried fecal samples (100 mg) from each animal were suspended in 100 µL of 0.1 MHCl and 1 mL of deionized water by vigorous mixing. The suspension was centrifuged at 13000 rpm for 10 min. The supernatant was processed using the solid-phase extraction (SPE) method, in which the sample was transferred to a previously activated column C-18 (Bond Elut C18, 1 mL, Agilent Technologies No. 12102001) and analyzed by high-performance liquid chromatography (HPLC; PerkinElmer Series N3896). The mobile phase used consisted of 80% solution A composed of NaH_2_PO_4_ (pH 2.2 using phosphoric acid, Sigma-Aldrich No. S8282-500G), and 20% solution B composed of acetonitrile (J.T. Baker).^36^ The detection threshold for standard curves was 100–5000 ppm for acetate (Sigma-Aldrich No. 45754), propionate (Sigma-Aldrich No. P1386), and butyrate (Sigma-Aldrich No. B103500), which were diluted in the same proportion as the mobile phase. The concentration of short-chain fatty acids determined in the samples were expressed in μg/mg fecal.

### Bacterial community profiling

#### Fecal sample collection, DNA extraction, and PCR amplification

DNA was extracted from 100 mg of feces from each animal using FavorPrep™ Stool DNA Isolation Mini Kit (Cat. FASTI 001-1, FAVORGEN© Biotech Corporation, Zhunan, Taiwan) according to the manufacturer's instructions. The purity of the DNA was assessed by the ratio of 260/280 nm absorbance using NanoDrop Lite Spectrophotometer (Thermo Scientific) equipment, and the integrity was evaluated by 0.5% agarose electrophoresis gel (100 volts, 50 min). The microbiota composition was carried out by sequencing the hypervariable region V3 (~281 base pair) of the 16S rRNA gene, which was amplified by endpoint PCR (PCR conditions in Supplementary Material Table S1) for each sample using specific primer complementary (forward V3-341F and reverse V3-518R) to the upstream and downstream regions of the locus of interest. The forward primer contains a known sequence barcode and a sequence with a 12-base pair Golay barcode that is different to identify each sample. The PCR product was visualized in 2% agarose gels, and the amount of the amplicon was measured by densitometry using the Image Lab v.4.1 software to ensure that the genomic library mixed equal amounts of amplicons. The genomic library was purified using a highly sensitive 2% agarose gel (E-GEL™ EX, 2%, Invitrogen™, Cat. G401002), and the concentration and size were evaluated with an Agilent 2100 Bioanalyzer Instrument (Agilent Technologies, Santa Clara, CA, USA). The PCR emulsion was carried out using Ion OneTouch™ 200 Template Kit v2 DL (Life Technologies, Carlsbad, CA, USA) according to the manufacturer's instructions.

#### Ion torrent next-generation sequencing

The semiconductor sequencing was made using the Ion 316 Chip Kit V2 Chip (Cat. 4488146, Life Technologies, Carlsbad, CA, USA) and an Ion Torrent PGM system v4.0.2. After sequencing, the reads obtained were filtered by PGM software to remove the polyclonal sequences (homopolymers > 6) and those with low quality (score ≤ 20). The filtered sequences were exported as FASTQ files, and the amplicon sequence variants (ASVs) were identified from reads that fulfilled the quality criteria using 2023.5 pipeline.^37^ Representative sequences were taxonomically assigned with the Greengenes2 database. The analysis was performed using RStudio 2025.05.0+496, and the data were imported into RStudio were using qiime2R 0.99.6 package and phyloseq 1.50.0 package to analyze microbial communities with relative abundance. The alpha diversity indices were computed based on the normalized ASV counts using the R package ‘Vegan’ to quantify the level of diversity within the sample, which was calculated with Chao1, ACE, Shannon, Simpson, and Fisher indices. The Chao1 and ACE indices reflected the taxa richness of a community. The Shannon and Simpson diversity (1 − D) indices reflect both species richness and evenness, where higher values represent greater species diversity and lower values indicate lower diversity, reflecting the dominance of a limited number of species. The Fisher index reflected the diversity of species. The beta diversity indices were plotted via principal coordinates analysis (PCoA). Differential abundance analysis was performed with DESEq2 1.46.0, the data were analyzed with tidyverse 2.0.0 and the figures were elaborated with ggplotify 0.1.2., and RColorBrewer 1.1-3.

### Statistical analysis

**Figure 1.**
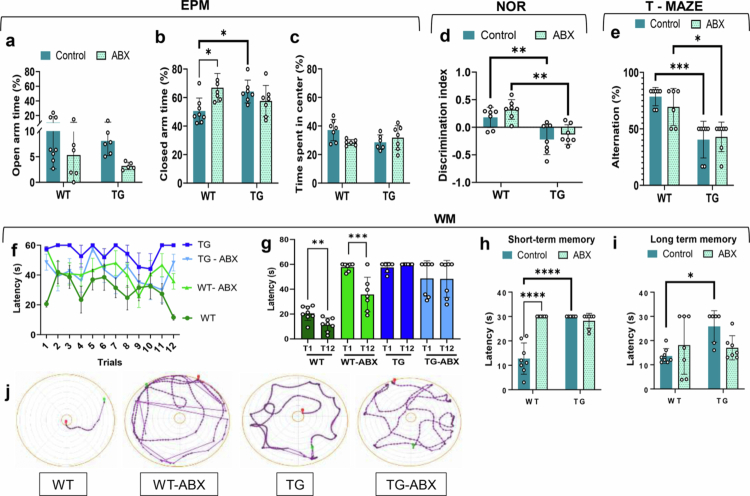
Behavioral evaluation of anxiety, learning, and memory. Elevated Plus Maze (EPM), Novel Object Recognition test (NOR), T-Maze, and Water Maze (WM) tests were used to evaluate behavioral and cognitive performance. EPM: a) The amount of time spent in the open arms was similar among the groups, b) The amount of time spent in the closed arms was directly proportional to the level of anxiety, c) The amount of time spent in the center was similar among the groups. NOR: d) The discrimination index (DI) was directly proportional to the recognition memory. T-maze: e) Working memory was directly proportional to the percentage of spontaneous alterations. WM: f) Spatial learning was inversely proportional to the latency to find the hidden platform. The x-axis represents 12 consecutive learning trials (T), g) Learning performance was compared between the first (T1) and the last trial (T12), h) Short-term spatial memory evaluation, i) Long-term spatial memory evaluation, j) Representative schemes of mice (WT, WT-ABX, TG, and TG-ABX) trajectories (dotted line and red balloon) from the star point (green balloon) to the platform area (central circle) in the WM. For (a–j), WT *n* = 8, TG *n* = 7, WT-ABX *n* = 7, and TG-ABX *n* = 7. The data are presented as mean ± SD bars. For (a–f), (h), and (i), two-way ANOVA with Tukey's post hoc correction was used. For (g) Student ' s *t*-test. Statistical significances are shown as *p < 0.05, **p < 0.01, ***p < 0.001, and ****p < 0.0001.

The data are expressed as the mean ± standard deviation (SD). Differences between experimental groups were compared by two-way analysis of variance (ANOVA) followed by Tukey ' s post hoc test, except for the learning performance between trials 1 and 2 in the WM test (Figure 1g) and Aβ plaque quantification (Figure 3b–c), both of which were evaluated by Student's *t*-test. Statistical differences between WT *vs.* TG, WT *vs.* WT-ABX, and TG *vs.* TG-ABX are depicted in the graphs. Correlation analysis was performed by the Pearson correlation coefficient. The analysis of microbial communities with relative abundance was performed by metacore algorithms and R (v3.6.0) in R-Studio 2025.05.0 + 496 software. Differences in taxa enrichment and the abundance of sequences were evidenced by DESeq2 and ANOVA. β diversity was analyzed by ANOSIM and ADONIS. Statistical analyses were performed with GraphPad Prism (v9.3.0). All the results were considered statistically significant at *p* < 0.05.

## Results

### Behavioral and cognitive alterations in female TG and antibiotic-treated WT mice

We evaluated behavioral and cognitive alterations in 6-month-old WT and TG female mice with and without ABX treatment. Anxiety was evaluated by the EPM. There was a significant interaction between genotype and treatment in the closed arms [F (1, 25) = 10.44, *p* = 0.003] and a significant effect of genotype on the time spent in the open arms [F (1, 21) = 5.195, *p* = 0.033]. TG female mice spent 64.25% of their time in the closed arms of the EPM, whereas WT females spent 50.54%, indicating greater anxiety-like behavior in TG mice (*p* = 0.04). Furthermore, ABX-treated WT mice (WT-ABX) spent 66.88% of their time in the closed arms, compared to 50.54% in WT controls, demonstrating that antibiotic treatment led to high anxiety levels in WT-ABX mice (*p* = 0.0143). No significant differences were seen between TG and TG-ABX in the time spent in the open arms. Time spent in the open arms or in the center of the maze was similar among the groups (Figure 1a–c).

Recognition memory impairments were associated with genotype [F (1, 24) = 29.73, *p* = < 0.0001], as TG female mice showed a negative discrimination index (DI) that was significantly different from that of WT (*p* = 0.007). Similarly, TG-ABX female mice showed a negative and significantly different DI compared to WT-ABX mice (*p* = 0.002) (Figure 1d). Short-term working memory was evaluated by the T-maze test. Genotype had a significant impact on the percentage of spontaneous alternations [F (1, 23) = 37.14, *p* < 0.0001], as TG female mice showed a lower percentage of alternations (40.49%) compared to WT females (78.56%) (*p* = 0.0002), which indicates impaired working memory. The antibiotic treatment did not cause any further alternations in working memory in TG mice. However, TG-ABX female mice had a reduced percentage of alternation than WT-ABX mice (*p* = 0.0103) (Figure 1e). Spatial learning and memory were evaluated by the Morris water maze. The learning curves showed significant differences among groups [F (3, 286) = 24.58, *p* < 0.0001] (Figure 1f, statistical analysis in Supplementary Material Table S2), as effective learning was observed in WT (T1 > T12, *p* = 0.005) and WT-ABX female mice (T1 > T12, *p* = 0.0015) (Figure 1g). However, T1 latency was higher in WT-ABX than WT controls, which indicates that the antibiotic caused impairments in the initial learning of WT female mice. In TG and TG-ABX female mice, there was no improvement in the latency to find the hidden platform during the testing trials (T1 = T12) (Figure 1g). Short- and long-term spatial memory were also impaired in TG female mice. Short-term memory was assessed 5 min after the last training trial. A statistically significant interaction between genotype and treatment [F (1, 23) = 40.27, *p* < 0.0001] and a significant effect of genotype [both, F (1, 23) = 26.67, *p* < 0.0001] were observed. In comparison to WT, the female TG mice were not able to find the place where the platform was located (*p* < 0.0001), demonstrating a deficient short-term memory. Notably, WT-ABX females also did not find the location of the platform during the maximum testing time, showing a significant difference compared to WT controls (*p* < 0.0001) (Figure 1h). Twenty-four hours later, long-term memory was evaluated. A statistically significant interaction between genotype and treatment [F (1, 24) = 5.696, *p* = 0.02] further showed that TG female mice spent more time trying to find the place where the platform was located compared to WT female mice (*p* = 0.02) (Figure 1i). These data demonstrate that TG female mice develop functional impairments in different cognitive domains compared to WT mice. However, ABX treatment caused anxiety and short-term memory impairments in WT female mice.

### The estrous cycle and the estrobolome function are altered in TG female mice

**Figure 2.**
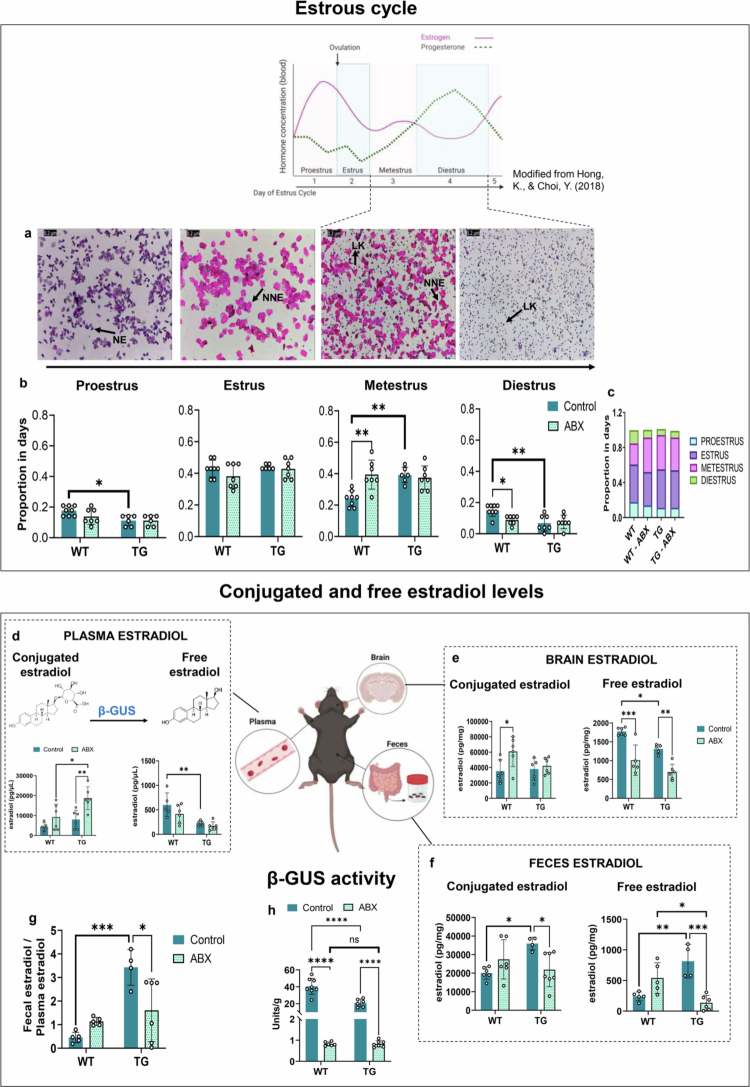
Estrous cycle, estradiol levels, and β-glucuronidase activity in TG female mice and antibiotic-treated WT mice. (a) Representative photomicrographs of each stage of the estrous cycle, showing the different types of cells: NE, nucleated epithelial cells (purple color); NNE, nonnucleated epithelial cells (pink color); and LK, leukocytes (purple dots), stained with hematoxylin‒eosin. b) The frequency of each estrous stage: Proestrus, estrus, metestrus, and diestrus in each experimental group. c) Proportion of the four estrous stages in all experimental groups. Conjugated (estradiol-17β-glucuronide) and free estradiol levels in plasma (d), brain (e), and fecal samples (f). g) Ratio of free estradiol levels in feces/plasma. (h) β-glucuronidase (β-GUS) enzymatic activity in fecal samples. For (a–c) WT *n* = 8, TG *n* = 7, WT-ABX *n* = 7, and TG-ABX *n* = 7. For (d) WT *n* = 5, TG *n* = 6, WT-ABX *n* = 6, and TG-ABX *n* = 6. For (e) WT *n* = 6, TG *n* = 5, WT-ABX *n* = 6, and TG-ABX *n* = 6. For (f) WT *n* = 6, TG *n* = 4, WT-ABX *n* = 6, and TG-ABX *n* = 7. For (h) WT *n* = 8, TG *n* = 6, WT-ABX *n* = 7, and TG-ABX *n* = 7. Two-way ANOVA with Tukey's post hoc correction. Statistical significances are shown as *p < 0.05, **p < 0.01, ***p < 0.001, and ****p < 0.0001.

In mice, the estrous cycle is divided into four stages: proestrus, estrus, metestrus, and diestrus, with a full cycle repeated every 4‒5 d (Figure 2a). We analyzed the proportion of estrous cycle stages for 28 d after careful classification of the vaginal cytology in each mouse. The proportion of estrous stages showed significant differences between TG and WT. There was a significant effect of genotype in the frequency of proestrus [F (1, 23) = 9.191, *p* = 0.005], with female TG mice having a lower proportion than WT females (*p* = 0.025). Estrus frequency showed similar values among the groups. In contrast, a significant interaction between genotype and treatment [F (1, 24) = 10.54, *p* = 0.003], an effect of genotype [F (1, 24) = 6.174, *p* = 0.0203], and treatment [F (1, 24) = 6.680, *p* = 0.0163] was observed in the proportion of metestrus. Female TG mice had a higher proportion of metestrus than WT mice (*p* = 0.0026), but also WT-ABX females showed higher values than WT controls (*p* = 0.0014). The proportion of diestrus showed a significant interaction between genotype and treatment [F (1, 25) = 5.238, *p* = 0.0308] and an effect of genotype [F (1, 25) = 9.807, *p* = 0.0044], as TG female mice had a lower proportion of diestrus than WT mice (*p* = 0.0034), but also WT-ABX females showed lower values than WT controls (*p* = 0.0320) (Figure 2b). A schematic representation showing the proportion of all estrous stages is depicted in Figure 2c.

Estradiol levels fluctuate during the estrous cycle, being higher during proestrus and lower during metestrus/diestrus. As mentioned before, estradiol can be metabolized in the liver through methylation, glucuronidation, or sulfonation to enable its excretion in bile.^38^ The gut microbiota can metabolize conjugated estrogens through sulfate ester hydrolysis^39^ or removal of the glucuronide group by the *estrobolome.*^16^^,^^40^ Thus, we determined the levels of free and estradiol 17-β-D-glucuronide (conjugated estradiol) in the plasma, brain, and feces, as well as the activity of β-glucuronidase in fecal samples from all the animals. Blood samples were taken only during metestrus/diestrus to avoid the variability associated with the cyclic fluctuations of sex hormones. Conjugated estradiol levels in plasma showed a statistically significant interaction with genotype [F (1, 19) = 9.269, *p* = 0.0067] and with antibiotic treatment [F (1, 19) = 13.00, *p* = 0.0019]. There was a significant increase in TG-ABX mice compared to TG mice (*p* = 0.0094) and WT-ABX mice (*p* = 0.0226). Free estradiol levels in plasma showed a significant interaction with genotype [F (1,18) = 20.61, *p* = 0.0003], as TG females exhibited significantly lower levels compared to WT females (*p* = 0.008). Antibiotic-treated female mice (WT-ABX and TG-ABX) had lower free estradiol levels than their control groups, but without significant differences (Figure 2d).

In the brain, the levels of conjugated estradiol showed a significant effect of treatment [F (1, 19) = 5.470, *p* = 0.03], as increased levels were noticed in WT-ABX females than WT controls (*p* = 0.0407). In contrast, the levels of free estradiol in brain samples showed a significant effect of genotype [F (1, 18) = 15.14, *p* = 0.0011] and treatment [F (1, 18) = 47.56, *p* < 0.0001], with a significant reduction in TG vs. WT mice (*p* = 0.0199), WT *vs.* WT-ABX (*p* = 0.0002), TG *vs.* TG-ABX (*p* = 0.0020) (Figure 2e).

In feces, conjugated estradiol levels showed a significant interaction between factors [F (1, 19) = 10.44, *p* = 0.0044], with higher conjugated estradiol levels in feces from TG mice than WT mice (*p* = 0.0243), indicating that TG female mice excreted a higher concentration of conjugated estradiol compared to WT controls. Notably, TG-ABX female mice showed lower conjugated estradiol concentration than TG mice (*p* = 0.0432). Free estradiol levels in feces showed a significant interaction between factors [F (1, 16) = 33.07, *p* < 0.0001] and treatment [F (1, 16) = 4.961, *p* = 0.04]. Free estradiol levels in feces in TG mice were higher compared to WT mice (*p* = 0.0017), indicating that TG mice excrete more free estradiol than WT mice. WT-ABX mice showed higher excretion of free estradiol compared to TG-ABX (*p* = 0.0129), but TG-ABX mice showed lower free estradiol levels in feces than TG mice (*p* = 0.0002) (Figure 2f). Therefore, the rate of free estradiol excretion (estradiol in feces/estradiol in plasma) was higher in TG mice (3.432 ± 0.75) compared to WT mice (0.45 ± 0.22) (*p* = 0.0003) and TG-ABX (1.607 ± 1.33) (*p* = 0.0165) (Figure 2g).

To determine whether the estradiol excretion rate was related to *estrobolome* function, we determined β-glucuronidase activity in fecal samples (Figure 2h). There was a statistically significant interaction between genotype and treatment [F (1, 23) = 20.19, *p* = 0.0002] and a significant effect of genotype [F (1, 23) = 20.18, *p* = 0.0002] and treatment [F (1, 23) = 187.6, *p* < 0.0001] on β-GUS activity. Female TG mice had reduced β-GUS activity than WT female mice (*p* < 0.0001), and as expected, β-GUS activity decreased after ABX treatment in both, WT-ABX *vs.* WT (*p* < 0.0001) and TG-ABX *vs.* TG (*p* < 0.0001) (Figure 2h).

Given the reduced β-glucuronidase activity observed in TG female mice, we assessed the enzyme activity in 6-months-old WT and TG male mice (Supplementary Material Figure S3). Unlike in females, there were no significant differences in total estradiol levels in plasma (*p* = 0.0628), or fecal samples (*p* = 0.2217), nor β-GUS activity between males (Supplementary Material Figure S3).

To explore whether liver alterations were related to the estradiol glucuronidation rate in female TG and WT-ABX mice, we determined liver morphology by H&E staining and alanine aminotransferase (ALT) activity in plasma samples (Supplementary Figures 4S and 5S). Histological examination indicated that WT female mice presented a normal liver morphology, but TG female mice demonstrated the presence of intracellular and extracellular lipid vacuoles in the liver. No differences in ALT activity were detected among the groups. The levels of estradiol-glucuronide in plasma (conjugated estradiol) were similar between WT and TG female mice (Figure 2d). Therefore, female TG mice may present a mild hepatic steatosis without a significant damage to the hepatic glucuronidation rate.

### Antibiotic treatment reduces the number of amyloid-β plaques in specific brain regions of female TG mice

**Figure 3.**
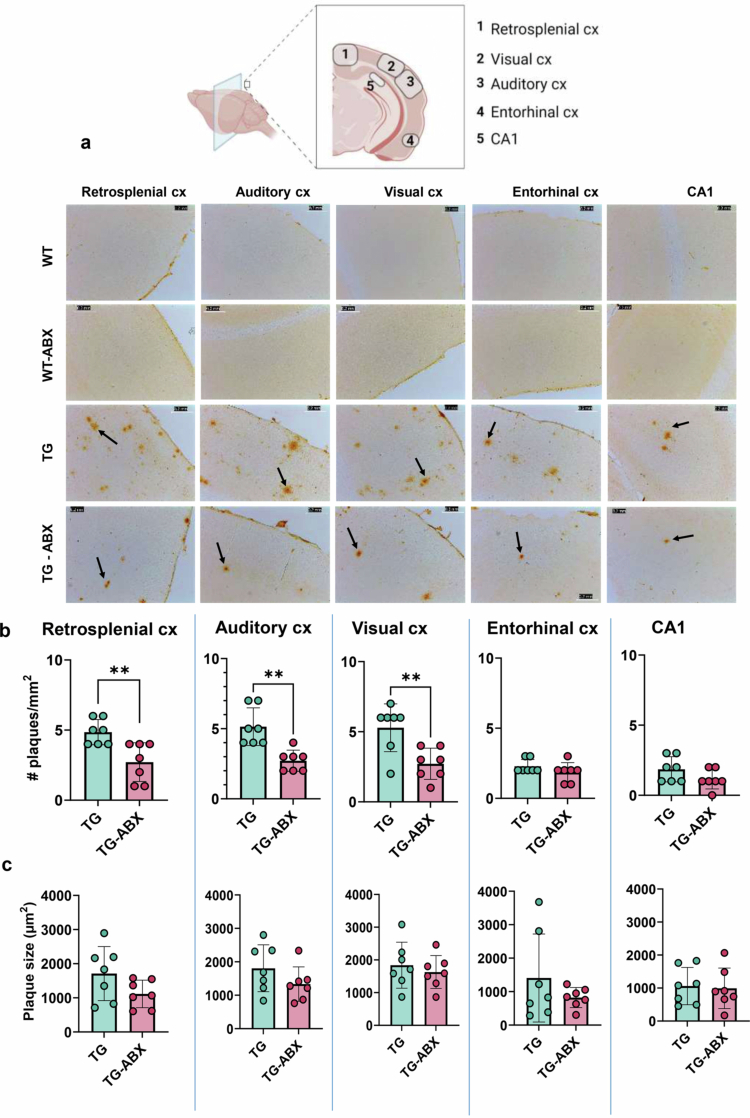
Antibiotics reduce the Aβ load in the brain of female TG mice. a) Representative images of Aβ plaques in the restrosplenial, auditory, visual, and entorhinal cortices and the CA1 hippocampal region of TG and TG-ABX. Representative images of WT and WT-ABX are shown to demonstrate the absence of Aβ aggregates. Aβ plaques are identified by black arrows. The scale bar equals 0.2 mm. b) Quantification of Aβ plaques. c) Size of Aβ plaques. For (a–c), WT *n* = 8, TG *n* = 7, WT-ABX *n* = 7, and TG-ABX *n* = 7. The data are presented as mean ± SD bars. Student's *t*-test. Statistical significances are shown as *p < 0.05, **p < 0.01, ***p < 0.001, and ****p < 0.0001.

Previous studies have shown that ABX treatment reduces amyloid-β plaques in the hippocampus and entorhinal cortex of 6-month-old male APP/PS1 mice^24^ but not in the cortical areas of 7-week-old and 3-month-old female APP/PS1-21 mice.^39^ We analyzed the amount and size of amyloid-β plaques in the retrosplenial, auditory, visual, and entorhinal cortices and CA1 hippocampal region of the 6-month-old female TG groups (Figure 3a). TG-ABX mice showed a significant decrease in the number of plaques in the retrosplenial, auditory, and visual cortices compared to the TG mice [(*t* = 3.441, df = 12, *p* = 0.0049), (*t* = 4.164, df = 12, *p* = 0.0013), and (*t* = 3.343, df = 12, *p* = 0.0059), respectively]. The entorhinal cortex and the CA1 hippocampal region had similar number of amyloid-β plaques in TG and TG-ABX mice (Figure 3b). Despite the reduced number of amyloid-β plaques in the retrosplenial, auditory, and visual cortices in TG-ABX female mice, the size of the plaques was not different in any brain region analyzed compared to TG mice (Figure 3c).

### Fecal production of short-chain fatty acids is affected by genotype and antibiotic treatment

**Figure 4.**
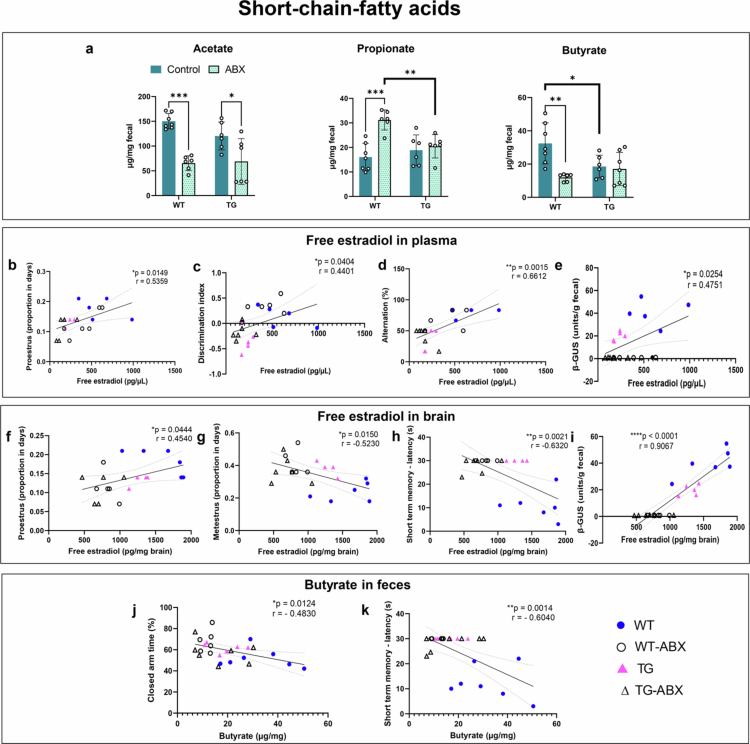
Alterations in the production of short-chain fatty acids in WT and TG female mice, treated or not with antibiotics. a) Acetate, propionate, and butyrate concentrations in fecal samples. (b–e) Correlation analysis of free estradiol in plasma with (b) the frequency of proestrus; (c) the discrimination index in NOR test; (d) the percentage of alternation in the T maze test; and (e) β-glucuronidase activity in fecal samples. (f–i) Correlation analysis of free estradiol in the brain with (f) the frequency of proestrus; (g) the frequency of metestrus; (h) latency to find the place where the platform was located in the short-term memory test; and (i) β-glucuronidase activity in fecal samples. Butyrate concentrations were negatively correlated with the time spent in enclosed arms in the EPM test (j) and with the latency to find the place where the platform was located in the short-term memory test (k). For (a), WT *n* = 6, TG *n* = 6, WT-ABX *n* = 6, and TG-ABX *n* = 7. Two-way ANOVA with Tukey's post hoc correction. Statistical significances are shown as *p < 0.05, **p < 0.01, ***p < 0.001, and ****p < 0.0001.

An important role of the gut microbiota is the fermentation of dietary carbohydrates to generate short-chain fatty acids (SCFAs), which can reach the brain.^41^^,^^42^ We measured the most predominant SCFAs produced by the gut microbiota:^43^ acetate (C2), propionate (C3), and butyrate (C4) in all experimental groups. The acetate concentration showed a statistically significant effect on the antibiotic treatment [F (1, 21) = 35.21, *p* < 0.0001], as WT-ABX and TG-ABX presented a reduced concentration compared to their control groups (*p* = 0.0002 and *p* = 0.0250, respectively). The propionate concentration showed a significant interaction between genotype and treatment [F (1, 21) = 10.31, *p* = 0.0042] and a significant effect of treatment [F (1, 21) = 15.71, *p* = 0.0007], as WT-ABX presented higher propionate concentrations than WT controls (*p* = 0.0002) and TG-ABX (*p* = 0.01). Finally, the butyrate concentration showed a statistically significant interaction between genotype and treatment [F (1, 22) = 7.237, *p* = 0.01] and a significant effect of treatment [F (1, 22) = 9.630, *p* = 0.005], as female TG mice presented lower butyrate concentrations than WT controls (*p* = 0.04), and antibiotic treatment reduced butyrate in WT animals (WT-ABX *vs.* WT, *p* = 0.0025) (Figure 4a).

### Free estradiol in plasma and brain correlates with β-GUS activity, estrous stages and cognitive function

Correlation analysis was run to determine the potential interaction between free estradiol levels in plasma and the brain, the estrous cycle, cognitive function, and β-glucuronidase activity in our experimental groups. In plasma, a positive correlation was observed between free estradiol levels and the proportion of proestrus (*r* = 0.5359, *p* = 0.0149) (Figure 4b), discrimination index in NOR test (*r* = 0.4401, *p* = 0.0404) (Figure 4c), percentage of spontaneous alterations (*r* = 0.6612, *p* = 0.0015) (Figure 4d), and β-glucuronidase activity (*r* = 0.4751, *p* = 0.0254) (Figure 4e). In the brain, free estradiol levels also showed a positive correlation with proestrus (*r* = 0.4540, *p* = 0.0444) (Figure 4f) and β-glucuronidase activity (*r* = 0.9067, *p* = < 0.0001) (Figure 4i), but a negative interaction was observed with metestrus (*r* = −0.5230, *p* = 0.0150) (Figure 4g), and the latency to find the site where the platform was located in the short-term memory test (*r* = −0.6320, *p* = 0.0021) (Figure 4h).

### Short-chain fatty acids and cognitive function

Correlation analysis was run to determine the potential interaction between short-chain fatty acids and cognitive function. Butyrate levels in fecal samples showed a negative interaction with the time spent in the closed arms of the EPM (*r* = −0.4830, *p* = 0.0124) (Figure 4j) and with the latency to find the site where the platform was located in the short-term memory test (*r* = −0.6040, *p* = 0.0014) (Figure 4k).

### Gut microbiota dysbiosis in female TG mice and WT-ABX mice associates with higher estradiol excretion

**Figure 5.**
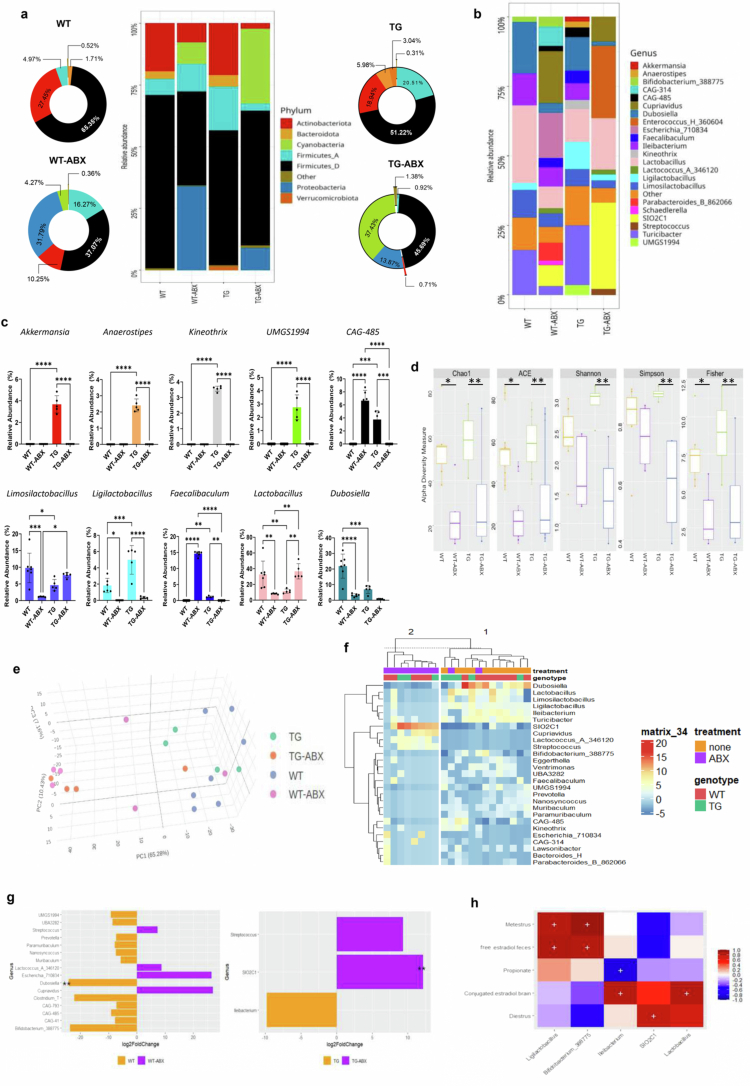
Gut microbiota profile and correlation analysis. a) Histogram of the relative abundance of Phyla (with a relative abundance greater than 1%). The pie chart shows the percentage of the most abundant taxa. Others refer to Phyla < 0.1% of relative abundance. Comparisons were performed by one-way ANOVA (Supplementary Material Table S3). b) Histogram of Genus' relative abundance (with a relative abundance greater than 1%) (Supplementary Material Table S4). c) The graphs on the right-side show the relative abundance of prominent bacterial genus that show group differences. Comparisons were performed by one-way ANOVA. d) Alpha diversity analyses indicated a decreased microbial load in the groups treated with antibiotics for 1 month. The Y-axis indicates the alpha diversity, and the X-axis represents the experimental groups. Comparisons were performed by one-way ANOVA (Supplementary Material Table S5), and the asterisks in the graph indicate *q*-values < 0.05. e) Beta diversity analyses (difference in global microbiota composition between groups) plotted as principal coordinates (PCoA) performed on Aitchison distances. The percentages on the axes of PCoA plots indicated the proportion of variation explained by the axis. Each point in the graph represents a sample. f) Heatmap of the differential abundance of significant amplicon sequence variants (ASVs) in the experimental groups. g) Differential abundance analysis of bacterial genera with DESeq2. The log_2_ fold change is shown by the horizontal bars. h) Spearman correlation analysis between the gut microbiota and measured biological variables. Red squares indicate significant positive correlation, and blue squares indicate significant negative correlation. Data represent the means ± SEM; statistical significances are shown as *p < 0.05, **p < 0.01, ***p < 0.001, ****p < 0.0001, two-way ANOVA. For (a–h), WT *n* = 7, TG *n* = 5, WT-ABX *n* = 6, and TG-ABX *n* = 5.

We aimed to identify disease-dependent bacterial taxa related to the phenotype of female TG mice and to elucidate whether specific bacteria play a role in *estrobolome* function. The bacterial composition of the gut microbiota was determined through massive sequencing of V3-16S rDNA libraries. The results were close to 4 million reads, with a mean of approximately 60,000 reads per sample. At the phylum level, there were statistical differences among the groups: female TG mice had a lower relative abundance of Firmicutes D (51.22%) compared to WT mice (65.35%) (*p* = 0.0014). In addition, Bacteroidota showed higher values in female TG mice than in WT mice (5.98% and 1.71%, respectively) (*p* = 0.3385), but Bacteroidota was not present in mice with antibiotic treatment (WT-ABX and TG-ABX). Antibiotic treatment reduces the relative abundance of Firmicutes A in TG (0.92%), but it is increased in WT mice (14.11%) (*p* = 0.0185). However, Firmicutes D was reduced in WT-ABX (32.15%) compared with WT control (65.35%) (*p* = 0.0100). Verrucomicrobiota appeared only in TG mice with a low relative abundance (3.04%). Notably, in female WT-ABX, there was a severe increase in Proteobacteria (27.58%) (Figure 5a and statistical analysis in Supplementary Material Table S3).

At the genus level, we noticed that female TG mice had some specific bacteria that were absent in the other experimental groups, such as *Akkermansia* (3.67%), *Anaerostipes* (1.81%)*, Kineothrix* (3.57%), and *UMGS1994* (2.75%). TG female mice also showed a higher relative abundance of *CAG-485* (3.76%) (*p* = 0.0001), *Fecalibaculum* (1.18%) (*p* = 0.0027), and *Ligilactobacillus* (4.98%) (*p* = 0.0009) compared to WT mice. In contrast, TG mice had a reduced relative abundance of *Limosilactobacillus* (4.65%) (*p* = 0.0143), *Lactobacillus* (11.45%) (*p* = 0.0047), and *Duboisella* (6.94%) (*p* = 0.0005) than the WT control group (9.80%, 32.69%, and 21.89%, respectively). On the other hand, antibiotic treatment causes a depletion of some taxa in TG-ABX mice, such as *Akkermansia* (0%) (*p* < 0.0001), *Anaerostipes* (0%) (*p* < 0.0001), *Kineothrix* (0%) (*p* < 0.0001), *UMGS1994* (0%) (*p* < 0.0001), *CAG-485* (0%) (*p* = 0.0002), *Limosilactobacillus* (7.64%) (*p* = 0.2368), *Ligilactobacillus* (0.34%) *(p* < 0.0001), *Faecalibaculum* (0%) (*p* = 0.0036), and *Dubosiella* (0.71%) (*p* = 0.1557) compared with TG female mice. In contrast, antibiotic treatment of WT-ABX females causes a greater abundance of CAG-485 (6.64% *vs*. 0%) (*p* < 0.0001), *Faecalibaculum* (14.64% *vs.* 0%) (*p* < 0.0001), but a reduced relative abundance of *Limosilactobacillus* (1.16% *vs*. 9.80%) (*p* = 0.0006), *Ligilactobacillus* (0% *vs*. 1.83) (*p* = 0.0232), *Lactobacillus* (8.16% *vs*. *p* = 0.0057) and *Dubosiella* (3.22% *vs.* 21.89%) (*p* < 0.0001) compared to WT mice (Figure 5b-c, and the statistical analysis in the Supplementary Material Table S4).

Alpha diversity analysis revealed significant differences between the groups. Chao1 and ACE indices indicated a reduction in microbial richness in the antibiotic-treated groups (WT-ABX and TG-ABX) compared with the control groups (WT and TG). Similarly, the Shannon and Simpson diversity indices revealed lower microbial diversity after antibiotic treatment, and Fisher's index supported these findings, showing an overall decrease in species diversity (Figure 5d and statistical analysis in Supplementary Material Table S5). Clustering the bacterial communities using principal coordinates analysis (PCoA) revealed a segregation between the groups, with a notable distance between the antibiotic treatment groups (WT-ABX and TG-ABX groups) and the control groups (WT and TG) (Figure 5e). Amplicon sequence variant (ASV) abundance analysis identified enrichments in the experimental groups. *Dubosiella* was significantly enriched in the WT control group, while in the antibiotic-treated TG mice *SIO2C1* genera was enriched (Figure 5f). The analysis of DESeq2 using ASV data indicated that bacterial diversity in WT-ABX was characterized by increased abundance of *Escherichia_710834*, *Cupriavidus,* and *Lactococcus_A_346120* and decreased abundance of *Dubosiella* (*p* < 0.01), *Bifidobacterium_388775*, and *Clostridium_T* in comparison with those in WT mice. The bacterial diversity of TG-ABX was characterized by an increased abundance of *SIO2C1* (*p* < 0.01) and *Streptococcus* and a decreased abundance of *Ilebacterium* (Figure 5g).

Spearman correlation analysis was used to represent statistically significant correlation values between taxa in the multivariate analysis and the measured features. *Ligilactobacillus* and *Bifidobacterium_388775* are associated with higher excretion of free estradiol and higher frequency of metestrus. *Ilebacterium* and *Lactobacillus* are associated with the levels of conjugated estradiol in the brain. *SIO2C1* abundance is related to a higher frequency of the diestrus stage (Figure 5h).

## Discussion

AD pathology in the brain begins decades before the clinical symptoms manifest. In women, this initial stage may occur during the reproductive period,^44^ even before the ovarian function ceases (perimenopause). Therefore, understanding the factors that contribute to the onset of AD pathology during the reproductive period in females is essential.

Several years ago, AD was thought to affect only the brain. However, recent research has revealed that peripheric alterations also play a significant role in the disease onset.^45^ The connection between the gut microbiota and AD has become increasingly important, as gut dysbiosis is observed in both symptomatic^46^^,^^47^ and asymptomatic AD patients.^48^ Despite the numerous studies focusing on gut microbiota alterations in AD pathology, there has been limited research on the potential consequences of gut dysbiosis, specifically in females.

The gut microbiota plays a vital role in modulating the circulating estrogen concentration through the *estrobolome.*^14^ Estrogens are important regulators of brain function, particularly in women.^49^ Gut dysbiosis and low estrogen bioavailability may be factors implicated in women's vulnerability to develop AD.

In this study, we aimed to describe the possible associations between the gut microbiota, estrogen bioavailability, and cognitive function using a transgenic mouse model. APP/PS1 mice exhibit impairments in spatial, working, and recognition memory starting at three months of age.^50^ Additionally, they show gut microbiota alterations before the formation of amyloid plaques (>3 months of age).^51^

Our results show that spontaneous gut dysbiosis in 6-month-old female TG mice is characterized by an altered Bacteroidota/Firmicutes ratio (Figure 5a) and a decreased abundance of *Limosilactobacillus, Lactobacillus, Faecalibaculum,* and *Dubosiella,* among others, compared to WT female mice (Figure 5c). In particular, *Lactobacillus* is well-known for its high β-glucuronidase activity,^13^^,^^52^ while the *gusA* gene has been cloned from *Limosilactobacillus gasseri* (formerly *Lactobacillus gasseri).*^53^ The reduction in those β-glucuronidase expressing bacteria parallels the reduced enzymatic activity in fecal samples, associated with lower free estradiol plasma levels, and higher free estradiol excretion rate in TG female mice compared to their WT littermates (Figure 2). Recent studies have shown that *Ligilactobacillus salivary* can degrade and conjugate estrogens,^54^ reducing their bioavailability and promoting their excretion. Our Spearman correlation analysis revealed that *Ligilactobacillus* promotes a higher excretion of free estradiol (Figure 5h), and we also observed higher conjugated-estradiol levels in the feces of TG female mice than in those of WT controls (Figure 2f). Therefore, our data in TG female mice indicate that gut dysbiosis promotes, on the one hand, lower estradiol-glucuronide deconjugation and, on the other hand, higher estradiol excretion.

We disrupted the function of the *estrobolome* by altering the gut microbiota through long-term antibiotic treatment in WT female mice. Previous studies have indicated that vancomycin, one of the antibiotics used in this study, eliminates β-glucuronidase activity in rodents,^55^^,^^56^ while ampicillin administration leads to higher estriol excretion in pregnant women.^57^ Our results show an effective alteration in the gut microbiota profile in WT female mice by the use of antibiotics (Figure 5). Notably, a significant reduction of *Limosilactobacillus* and *Lactobacillus* was found in WT-ABX compared to WT mice, the same bacteria strains associated with the dysfunctional *estrobolome* in TG mice. Therefore, our findings align with earlier reports, confirming that antibiotic treatment depletes β-glucuronidase activity through targeted elimination of β-glucuronidase-producing bacteria (i.e., *Lactobacillus* and *Limosilactobacillus*).

At 6 months of age, female mice experience regular reproductive cycles,^58^ and ovulation typically occurs during proestrus/estrus.^27^ Reproductive senescence in rodents begins around 9 months of age, and it is marked by prolonged metestrus and sustained diestrus.^59^ Here, we observed that 6-month-old TG female mice already had an increased frequency of metestrus (an estrous stage characterized by low estrogen production^60^^,^^61^) and a decreased frequency of proestrus. WT-ABX female mice also had an increased number of days in metestrus, increasing the number of anovulatory days, similar to TG female mice (Figure 2b). The longer the metestrus, the lower the fertility window, mirroring the onset of reproductive senescence. Hence, the dysbiotic TG and WT-ABX female groups exhibited a premature reproductive senescence profile compared to WT eubiotic mice. To explore if, besides the gut dysbiosis, alterations in hepatic function may also be implicated in estrobolome alterations, we measured alanine aminotransferase (ALT) activity in plasma samples from both WT and TG female mice. No significant differences were found between the two groups (see Supplementary Figure 5S). Additionally, we examined liver morphology using H&E staining in 6-month-old WT and TG female mice. TG female mice showed the presence of intracellular lipid droplets, which were not observed in WT mice (see Supplementary Figure 4S). However, comparing the plasma levels of estradiol 17-β-D-glucuronide (produced by hepatic conjugation), there were no significant differences between WT and TG female mice (Figure 2d). These findings suggest that TG female mice exhibit a mild hepatic steatosis without significant functional damage or alterations in the hepatic glucuronidation rate. Therefore, the observed gut dysbiosis and decreased β-glucuronidase activity in TG female mice may be contributing factors associated with the reduced free estradiol bioavailability and increased estradiol excretion rate.

Estradiol is a key hormone for memory function, especially in females.^62^ In our study, TG female mice had lower free estradiol levels in the brain and impairments in several cognitive domains (working, recognition, and spatial memory) compared to WT mice. Moreover, antibiotic treated WT mice (WT-ABX) also had reduced levels of free estradiol in the brain and demonstrate an impaired short-term spatial memory and increased anxiety compared to WT mice (Figure 1). Correlation analysis indicates that lower circulating levels of free estradiol positively correlated with impairments in recognition and working memory, and lower levels of free estradiol in the brain were associated with short-term memory impairments in both WT and TG mice (Figure 4b). A similar antibiotic treatment was previously used in male C57BL/6N^63^ and APP/PS1 mice,^24^ resulting in cognitive impairments.

Several studies have shown that antibiotic treatment can lead to changes in behavior and cellular alterations in the brains of various mouse models.^64-67^ To better understand the direct effects of antibiotics on neuronal and cognitive functions, independent of any changes to the microbiota, it is essential to use germ-free mouse models.^68^ In our current study, antibiotic-treated female mice exhibited lower levels of free estradiol in the brain compared to their control counterparts (see Figure 2e). The behavioral changes observed in WT-ABX mice may be linked to the direct effects of antibiotics on neuronal estrogen production. However, we were unable to conduct germ-free mouse experiments to assess the direct effect of antibiotic treatment on neuronal estrogen production, independent of gut microbiota modulation. Additional research is essential to understand the impact of antibiotics on brain and body functions, independent of the gut microbiota, as these effects are complex and require thorough characterization. In addition, antibiotics might impact the excretion rate of other estrogen metabolites (i.e., sulfated estrogens), which are broken down by gut bacteria.^39^^,^^69^ We focused on glucuronide estrogens, as they are targeted by the β-glucuronidase enzyme. However, it is presumed that antibiotic treatment may also affect methylated or sulfonated estrogen metabolites. On the other hand, TG-ABX female mice did not present any further cognitive deterioration, despite showing lower free estradiol levels in the brain and a decreased size of β-amyloid plaques in specific brain regions compared to TG females. Previous research has indicated that both short- or long-term antibiotic treatment had no noticeable effects on the overall density of β-amyloid plaques on female APP/PS1,^39^^,^^70^ despite a profound amyloid pathology as they aged.^71^^,^^72^ However, those studies determined the impact of antibiotic treatment on amyloid burden across the entire cortex, and here, we evaluated amyloid pathology in specific cortical regions. A reduction in the number of β-amyloid plaques in the retrosplenial, auditory, and visual cortices was observed after one month of antibiotic treatment in TG-ABX female mice (Figure 3). However, the TG-ABX group did not show improvements in behavior compared to TG mice (Figure 1). Studies have suggested a positive correlation between the presence of β-amyloid plaques and cognitive decline in the early stages of the disease in humans.^64^ The reductions in β-amyloid plaques observed in the retrosplenial, auditory and visual cortices in TG-ABX mice but not in the CA1 region of the hippocampus (a key structure for learning and memory^66^) may be a condition that may relate to the lack of cognitive improvement following ABX treatment in TG mice. Importantly, however, TG-ABX female mice presented lower estradiol levels in the brain than TG mice. It is known that ovariectomy, which leads to a complete depletion of estrogen production, exacerbates amyloid deposition in the brain of female 5xTGAD mice.^73^ Hence, reducing brain estrogen levels in TG-ABX mice may actually increase the amyloid plaque load. We may speculate that amyloid pathology responds differently to depletions of the gut microbiota (by antibiotic treatment) than to estrogen disposition/production in the brain. In our current study, both conditions were present in TG-ABX female mice, resulting in a selective regional β-amyloid plaque reduction but without significant changes in spatial and working memory scores. It would have been crucial to examine the presence of soluble β-amyloids rather than just the larger amyloid plaques in TG mice, as antibiotics may impair soluble forms of Aβ and plaque aggregation. A recent study demonstrated that fecal transplantation of young to aged zebrafish restores *estrobolome* dysfunction induced by a toxic pollutant.^74^ Future studies should implement fecal microbiota transplantation from WT donors to TG mice to further determine whether changes in microbial communities directly impact estrogen levels and cognitive function in AD mice.

One of the primary functions of gut bacteria is to metabolize dietary fiber, resulting in the production of SCFAs as bioproducts,^67^ such as butyrate, a powerful neuromodulator.^68^^,^^69^ In our observations, we found that butyrate production was lower in TG female mice compared to WT controls (Figure 4a). This reduction may be attributed to a decrease in the abundance of Firmicutes, which comprises most of butyrate-producing bacteria. Additionally, butyrate production was significantly reduced in WT-ABX compared to WT controls. The fecal butyrate concentration was found to have a negative correlation with anxiety levels and short-term memory impairments (Figure 4j). Previous studies have demonstrated that fecal butyrate levels decline in female 3xTGAD mice as they age.^75^ Therefore, we confirmed that AD phenotype in TG mice is characterized by reduced butyrate production, while antibiotic treatment diminishes the butyrate concentration also in WT female mice. Acetate and propionate levels were not significantly different between WT and TG female mice. However, antibiotic treatment resulted in a significant depletion of specific taxa, leading to a marked reduction in acetate in both WT and TG mice. In contrast, propionate levels were elevated in WT-ABX mice compared to WT controls (Figure 4a). Our previous research demonstrated that fecal propionate levels are related to brain propionate levels in nine-month-old 3xTgAD female mice.^75^ Propionate induces prominent metabolic dysfunction in astrocytes and increases neuroinflammation.^24^ Therefore, alterations in short-chain fatty acids in TG and WT-ABX mice may be partially linked to the behavioral and cognitive impairments observed in these animals.

In summary, our current study demonstrates that spontaneous gut dysbiosis in TG female mice results in an altered *estrobolome* function, which is associated with a low estrogen bioavailability. In addition, gut dysbiosis in TG female mice results in lower butyrate production. Gut dysbiosis may contribute to cognitive impairments and early reproductive senescence in 6-month-old TG female mice. We also found that antibiotic-induced gut dysbiosis in WT female mice is associated with a decreased estradiol availability and impairments in short-term memory. A decreased abundance of *Lactobacillus* and *Limosilacobacillus* in both WT-ABX and TG female mice, and an increased abundance of *Ligilactobacillus* in TG mice were associated with a higher estradiol excretion rate and lower estradiol concentrations in the brain.

Depriving the brain of estrogen's neuroprotective actions may cause a premature neuronal failure, particularly in females. Therefore, preventing gut dysbiosis and maintaining a healthy *estrobolome* may be crucial for women to reduce their risk of developing dementia in older age.^76-81^

## Supplementary Material

Supplementary material Supplementary_files_edited clean

## Data Availability

All sequencing data generated in this study are deposited at NCBI sequence read archive repository as bioproject ID: PRJNA1266227 (http://www.ncbi.nlm.nih.gov/bioproject/1266227). Any additional information reported in this paper is available from the lead contact upon request.

## Data deposition

https://figshare.com/s/dc43a8c9ede6efd8c17a?file=55095179.

## References

[1] Jack CR, Andrews JS, Beach TG, Buracchio T, Dunn B, Graf A, Hansson O, Ho C, Jagust W, McDade E, et al. Revised criteria for diagnosis and staging of Alzheimer's disease: Alzheimer's Association Workgroup. Alzheimers Dement. 2024;20:5143–5169. doi: 10.1002/alz.13859. 38934362 PMC11350039

[2] 2020 Alzheimer's disease facts and figures. Alzheimers Dement. 2020;16:391–460. doi: 10.1002/alz.12068. 32157811

[3] Pike CJ. Sex and the development of Alzheimer's disease. J Neurosci Res. 2017;95:671–680. doi: 10.1002/jnr.23827. 27870425 PMC5120614

[4] Scheyer O, Rahman A, Hristov H, Berkowitz C, Isaacson R, Diaz Brinton R, Mosconi L. Female sex and Alzheimer's risk: the menopause connection. J Prev Alzheimers Dis. 2018;5:225–230. doi: 10.14283/jpad.2018.34. 30298180 PMC6198681

[5] Mosconi L, Berti V, Guyara-Quinn C, McHugh P, Petrongolo G, Osorio RS, Connaughty C, Pupi A, Vallabhajosula S, Isaacson RS, et al. Perimenopause and emergence of an Alzheimer's bioenergetic phenotype in brain and periphery. PLoS One. 2017;12:e0185926. doi: 10.1371/journal.pone.0185926. 29016679 PMC5634623

[6] Bansal S, Swami R, Chaudhary R, Mahendiratta S, Kaur H, Chopra K, Medhi B. Evidence-based neuroprotective potential of nonfeminizing estrogens: in vitro and in vivo studies. Eur J Neurosci. 2024;60:6046–6056. doi: 10.1111/ejn.16512. 39297873

[7] Rocca WA, Cotman CW. S1-01-02: cognitive impairment or dementia in women who underwent bilateral oophorectomy before menopause. Alzheimers Dement. 2010;6:S64. doi: 10.1016/j.jalz.2010.05.183.

[8] Phung TKT, Waltoft BL, Laursen TM, Settnes A, Kessing LV, Mortensen PB, Waldemar G. Hysterectomy, oophorectomy and risk of dementia: a nationwide historical cohort study. Dement Geriatr Cogn Disord. 2010;30:43–50. doi: 10.1159/000314681. 20689282

[9] Agca C, Klakotskaia D, Stopa EG, Schachtman TR, Agca Y. Ovariectomy influences cognition and markers of Alzheimer's disease. J Alzheimers Dis. 2020;73:529–541. doi: 10.3233/JAD-190935. 31796679

[10] Tao X, Yan M, Wang L, Zhou Y, Xia T, Liu X, Pan R, Chang Q. Effects of estrogen deprivation on memory and expression of related proteins in ovariectomized mice. Ann Transl Med. 2020;8:356. doi: 10.21037/atm.2020.02.57. 32355800 PMC7186664

[11] Yue X, Lu M, Lancaster T, Cao P, Honda S, Staufenbiel M, Harada N, Zhong Z, Shen Y, Li R. Brain estrogen deficiency accelerates Aβ plaque formation in an Alzheimer's disease animal model. Proc Natl Acad Sci U S A. 2005;102:19198–19203. doi: 10.1073/pnas.0505203102. 16365303 PMC1323154

[12] Ervin SM, Li H, Lim L, Roberts LR, Liang X, Mani S, Redinbo MR. Gut microbial β-glucuronidases reactivate estrogens as components of the estrobolome that reactivate estrogens. J Biol Chem. 2019;294:18586–18599. doi: 10.1074/jbc.RA119.010950. 31636122 PMC6901331

[13] Kwa M, Plottel CS, Blaser MJ, Adams S. The Intestinal Microbiome and Estrogen Receptor-Positive Female Breast Cancer. J Natl Cancer Inst. 2016;108(8):djw029. doi: 10.1093/jnci/djw029. 27107051 PMC5017946

[14] Hu S, Ding Q, Zhang W, Kang M, Ma J, Zhao L. Gut microbial beta-glucuronidase: a vital regulator in female estrogen metabolism. Gut Microbes. 2023;15:2236749. doi: 10.1080/19490976.2023.2236749. 37559394 PMC10416750

[15] Shin JH, Park Y, Sim M, Kim S, Joung H. Serum level of sex steroid hormone is associated with diversity and profiles of human gut microbiome. Res Microbiol. 2019;170:192–201. doi: 10.1016/j.resmic.2019.03.003. 30940469

[16] Flores R, Shi J, Fuhrman B, Xu X, Veenstra TD, Gail MH, Gajer P, Ravel J, Goedert JJ. Fecal microbial determinants of fecal and systemic estrogens and estrogen metabolites: a cross-sectional study. J Transl Med. 2012;10:253. doi: 10.1186/1479-5876-10-253. 23259758 PMC3552825

[17] Chen KL, Madak-Erdogan Z. Estrogen and microbiota crosstalk: should we pay attention? Trends Endocrinol Metab. 2016;27:752–755. doi: 10.1016/j.tem.2016.08.001. 27553057

[18] Vogt NM, Kerby RL, Dill-McFarland KA, Harding SJ, Merluzzi AP, Johnson SC, Carlsson CM, Asthana S, Zetterberg H, Blennow K, et al. Gut microbiome alterations in Alzheimer's disease. Sci Rep. 2017;7(1):13537. doi: 10.1038/s41598-017-13601-y. 29051531 PMC5648830

[19] Ling Z, Zhu M, Yan X, Cheng Y, Shao L, Liu X, Jiang R, Wu S. Structural and functional dysbiosis of fecal microbiota in Chinese patients with Alzheimer's disease. Front Cell Dev Biol. 2021;8:634069. doi: 10.3389/fcell.2020.634069. 33614635 PMC7889981

[20] Liu P, Wu L, Peng G, Han Y, Tang R, Ge J, Zhang L, Jia L, Yue S, Zhou K, et al. Altered microbiomes distinguish Alzheimer's disease from amnestic mild cognitive impairment and health in a Chinese cohort. Brain Behav Immun. 2019;80:633–643. doi: 10.1016/j.bbi.2019.05.008. 31063846

[21] Zhang L, Wang Y, Xiayu X, Shi C, Chen W, Song N, Fu X, Zhou R, Xu Y, Huang L, et al. Altered Gut microbiota in a mouse model of Alzheimer's disease. J Alzheimers Dis. 2017;60:1241–1257. doi: 10.3233/JAD-170020. 29036812

[22] Cuervo-Zanatta D, Garcia-Mena J, Perez-Cruz C. Gut microbiota alterations and cognitive impairment are sexually dissociated in a transgenic mice model of Alzheimer's disease. J Alzheimers Dis. 2021;82:S195–S214. doi: 10.3233/JAD-201367. 33492296

[23] Vikram A, Kim Y, Kumar S, Li Q, Kassan M, Jacobs JS, Irani K. Vascular microRNA-204 is remotely governed by the microbiome and impairs endothelium-dependent vasorelaxation by downregulating Sirtuin1. Nat Commun. 2016;7:12565. doi: 10.1038/ncomms12565. 27586459 PMC5025761

[24] Cuervo-Zanatta D, Syeda T, Sánchez-Valle V, Irene-Fierro M, Torres-Aguilar P, Torres-Ramos MA, Shibayama-Salas M, Silva-Olivares A, Noriega LG, Torres N, et al. Dietary fiber modulates the release of gut bacterial products preventing cognitive decline in an Alzheimer's mouse model. Cell Mol Neurobiol. 2023;43:1595–1618. doi: 10.1007/s10571-022-01268-7. 35953741 PMC11412426

[25] Byers SL, Wiles MV, Dunn SL, Taft RA. Mouse estrous cycle identification tool and images. PLoS One. 2012;7:e35538. doi: 10.1371/journal.pone.0035538. 22514749 PMC3325956

[26] Cora MC, Kooistra L, Travlos G. Vaginal cytology of the laboratory rat and mouse: review and criteria for the staging of the estrous cycle using stained vaginal smears. Toxicol Pathol. 2015;43:776–793. doi: 10.1177/0192623315570339. 25739587 PMC11504324

[27] Miller BH, Takahashi JS. Central circadian control of female reproductive function. Front Endocrinol (Lausanne). 2013;4:195. doi: 10.3389/fendo.2013.00195. 24478756 PMC3898595

[28] Walf AA, Frye CA. The use of the elevated plus maze as an assay of anxiety-related behavior in rodents. Nat Protoc. 2007;2:322–328. doi: 10.1038/nprot.2007.44. 17406592 PMC3623971

[29] Deacon RMJ, Rawlins JNP. T-maze alternation in the rodent. Nat Protoc. 2006;1:7–12. doi: 10.1038/nprot.2006.2. 17406205

[30] Lueptow LM. Novel object recognition test for the investigation of learning and memory in mice. J Vis Exp. 2017;2017:55718. doi: 10.3791/55718-v. PMC5614391 28892027

[31] Nunez J. Morris water maze experiment. J Vis Experiments. 2008;19:897. doi: 10.3791/897. PMC2872979 19066539

[32] Vorhees CV, Williams MT. Morris water maze: procedures for assessing spatial and related forms of learning and memory. Nat Protoc. 2006;1:848–858. doi: 10.1038/nprot.2006.116. 17406317 PMC2895266

[33] Paxinos G, Franklin K. The mouse brain in stereotaxic coordinates, compact|978-0-12-374244-5|. Elsevier; 2008. p. 827–828.

[34] Rodriguez-Callejas JD, Fuchs E, Perez-ruz C. Evidence of tau hyperphosphorylation and dystrophic microglia in the common marmoset. Front Aging Neurosci. 2016;8:315. doi: 10.3389/fnagi.2016.00315. 28066237 PMC5177639

[35] Nelson PT, Alafuzoff I, Bigio EH, Bouras C, Braak H, Cairns NJ, Castellani RJ, Crain BJ, Davies P, Tredici KD, et al. Correlation of Alzheimer disease neuropathologic changes with cognitive status: a review of the literature. J Neuropathol Exp Neurol. 2012;71:362–381. doi: 10.1097/NEN.0b013e31825018f7. 22487856 PMC3560290

[36] Reitman S, Frankel S. A colorimetric method for the determination of serum glutamic oxalacetic and glutamic pyruvic transaminases. Am J Clin Pathol. 1957;28:56–63. doi: 10.1093/ajcp/28.1.56. 13458125

[37] De Baere S, Eeckhaut V, Steppe M, De Maesschalck C, De Backer P, Van Immerseel F, Croubels S. Development of a HPLC–UV method for the quantitative determination of four short-chain fatty acids and lactic acid produced by intestinal bacteria during in vitro fermentation. J Pharm Biomed Anal. 2013;80:107–115. doi: 10.1016/j.jpba.2013.02.032. 23542733

[38] Duffel MW. Cytosolic sulfotransferases in endocrine disruption. Essays Biochem. 2024;68:541–553. doi: 10.1042/EBC20230101. 38699885 PMC11531609

[39] Van Eldere J, Robben J, De Pauw G, Merckx R, Eyssen H. Isolation and identification of intestinal steroid-desulfating bacteria from rats and humans. Appl Environ Microbiol. 1988;54:2112–2117. doi: 10.1128/aem.54.8.2112-2117.1988. 3178214 PMC202812

[40] Bolyen E, Rideout JR, Dillon MR, Bokulich NA, Abnet CC, Al-Ghalith GA, Alexander H, Alm EJ, Arumugam M, Asnicar F, et al. Reproducible, interactive, scalable and extensible microbiome data science using QIIME 2. Nat Biotechnol. 2019;37(8):852–857. doi: 10.1038/s41587-019-0209-9. 31341288 PMC7015180

[41] Neuman H, Debelius JW, Knight R, Koren O. Microbial endocrinology: the interplay between the microbiota and the endocrine system. FEMS Microbiol Rev. 2015;39:509–521. doi: 10.1093/femsre/fuu010. 25701044

[42] Dodiya HB, Kuntz T, Shaik SM, Baufeld C, Leibowitz J, Zhang X, Gottel N, Butovsky O, Gilbert JA, Sisodia SS. Sex-specific effects of microbiome perturbations on cerebral Ab amyloidosis and microglia phenotypes. J Exp Med. 2019;216:1542–1560. doi: 10.1084/jem.20182386. 31097468 PMC6605759

[43] Dalile B, Van Oudenhove L, Vervliet B, Verbeke K. The role of short-chain fatty acids in microbiota–gut–brain communication. Nat Rev Gastroenterol Hepatol. 2019;16:461–478. doi: 10.1038/s41575-019-0157-3. 31123355

[44] Mitchell RW, On NH, Del Bigio MR, Miller DW, Hatch GM. Fatty acid transport protein expression in human brain and potential role in fatty acid transport across human brain microvessel endothelial cells. J Neurochem. 2011;117:735–746. 21395585 10.1111/j.1471-4159.2011.07245.x

[45] Den Besten G, van Eunen K, Groen AK, Venema K, Reijngoud D, Bakker BM. The role of short-chain fatty acids in the interplay between diet, gut microbiota, and host energy metabolism. J Lipid Res. 2013;54:2325–2340. doi: 10.1194/jlr.R036012. 23821742 PMC3735932

[46] Liao H, Cheng J, Pan D, Deng Z, Liu Y, Jiang J, Cai J, He B, Lei M, Li H, et al. Association of earlier age at menopause with risk of incident dementia, brain structural indices and the potential mediators: a prospective community-based cohort study. EClinicalMedicine. 2023;60:102033. doi: 10.1016/j.eclinm.2023.102033. 37396803 PMC10314163

[47] Castillo X, Castro-Obregón S, Gutiérrez-Becker B, Gutiérrez-Ospina G, Karalis N, Khalil AA, Lopez-Noguerola JS, Rodríguez LL, Martínez-Martínez E, Perez-Cruz C, et al. Re-thinking the etiological framework of neurodegeneration. Front Neurosci. 2019;13:728. doi: 10.3389/fnins.2019.00728. 31396030 PMC6667555

[48] Haran JP, Bhattarai SK, Foley SE, Dutta P, Ward DV, Bucci V, McCormick BA, Pettigrew MM, Gilbert J, Faith J. Alzheimer's disease microbiome is associated with dysregulation of the anti-inflammatory P-glycoprotein pathway. mBio. 2019;10. doi: 10.1128/mBio.00632-19. PMC6509190 31064831

[49] Saha P, Sisodia SS. Role of the gut microbiome in mediating sex-specific differences in the pathophysiology of Alzheimer's disease. Neurotherapeutics. 2024;21:e00426. doi: 10.1016/j.neurot.2024.e00426. 39054179 PMC11585881

[50] Ferreiro AL, Choi J, Ryou J, Newcomer EP, Thompson R, Bollinger RM, Hall-Moore C, Ndao IM, Sax L, Benzinger TLS, et al. Gut microbiome composition may be an indicator of preclinical Alzheimer's disease. Sci Transl Med. 2023;15. doi: 10.1126/scitranslmed.abo2984. PMC10680783 37315112

[51] Hara Y, Waters EM, McEwen BS, Morrison JH. Estrogen effects on cognitive and synaptic health over the lifecourse. Physiol Rev. 2015;95:785–807. doi: 10.1152/physrev.00036.2014. 26109339 PMC4491541

[52] Bruce-Keller AJ, Gupta S, Knight AG, Beckett TL, McMullen JM, Davis PR, Murphy MP, Van Eldik LJ, St Clair D, Keller JN. Cognitive impairment in humanized APP×PS1 mice is linked to Aβ(1-42) and NOX activation. Neurobiol Dis. 2011;44:317–326. doi: 10.1016/j.nbd.2011.07.012. 21798347 PMC3185143

[53] Chen Y, Fang L, Zhou H, Fan Y, Lin L, Li J, Xu J, Ma Y, Racz B. Gut microbiome alterations precede cerebral amyloidosis and microglial pathology in a mouse model of Alzheimer's disease. Biomed Res Int. 2020;2020. doi: 10.1155/2020/8456596. PMC7273394 32596386

[54] Farkas S, Szabó A, Török B, Sólyomvári C, Fazekas CL, Bánrévi K, Correia P, Chaves T, Zelena D. Ovariectomy-induced hormone deprivation aggravates Aβ1-42 deposition in the basolateral amygdala and cholinergic fiber loss in the cortex but not cognitive behavioral symptoms in a triple transgenic mouse model of Alzheimer's disease. Front Endocrinol. 2022;13:985424. doi: 10.3389/fendo.2022.985424. PMC9596151 36303870

[55] Russell WM, Klaenhammer TR. Identification and Cloning of gusA, Encoding a New β-Glucuronidase from Lactobacillus gasseri ADH. Appl Environ Microbiol. 2001;67:1253–1261. doi: 10.1128/AEM.67.3.1253-1261.2001. 11229918 PMC92721

[56] Aragón A, Jurado R, Jara J, Rodríguez JM, Orgaz B. Investigating the metabolism of estrogens in ligilactobacillus salivarius strains isolated from human milk and vaginal microbiota. Nutrients. 2024;16:861. doi: 10.3390/nu16060861. 38542771 PMC10975273

[57] Taylor M, Flannigan K, Rahin H, Mohamud A, Lewis I, Hirota S, Greenway s. Vancomycin relieves mycophenolate mofetil–induced gastrointestinal toxicity by eliminating gut bacterial β-glucuronidase activity. Sci Adv. 2019;15:eaax2358. 10.1126/sciadv.aax2358 PMC6685722 31457102

[58] Zhang LT, Westblade LF, Iqbal F, Taylor MR, Chung A, Satlin MJ, Magruder M, Edusei E, Albakry S, Botticelli B, et al. Gut microbiota profiles and fecal beta-glucuronidase activity in kidney transplant recipients with and without post-transplant diarrhea. Clin Transplant. 2021;35:e14260. doi: 10.1111/ctr.14260. 33605497

[59] Martin F, Peltonen J, Laatikainen T, Pulkkinen M, Adlercreutz H. Excretion of progesteone metabolites and estriol in faeces from pregnant women during ampicillin administration. J Steroid Biochem. 1975;6:1339–1346. doi: 10.1016/0022-4731(75)90363-5. 1181489

[60] Bahougne T, Angelopoulou E, Jeandidier N, Simonneaux V. Individual evaluation of luteinizing hormone in aged C57BL/6 J female mice. Geroscience. 2019;42:323–331. doi: 10.1007/s11357-019-00104-z. 31641925 PMC7031456

[61] LeFevre J, McClintock MK. Reproductive senescence in female rats: a longitudinal study of individual differences in estrous cycles and behavior. Biol Reprod. 1988;38:780–789. doi: 10.1095/biolreprod38.4.780. 3401536

[62] Caligioni CS. Assessing reproductive status/stages in mice. Curr Protoc Neurosci. 2009; Appendix 4:Appendix 4I.. doi: 10.1002/0471142301.nsa04is48. PMC2755182 19575469

[63] Mroczyńska M, Libudzisz Z. β-glucuronidase and β-glucosidase activity of Lactobacillus and Enterococcus isolated from human feces. Pol J Microbiol. 2010;59:265–269. doi: 10.33073/pjm-2010-040. 21466044

[64] Wang S, Qu Y, Chang L, Pu Y, Zhang K, Hashimoto K. Antibiotic-induced microbiome depletion is associated with resilience in mice after chronic social defeat stress. J Affect Disord. 2020;260:448–457. doi: 10.1016/j.jad.2019.09.064. 31539679

[65] Wang P, Tu K, Cao P, Yang Y, Zhang H, Qiu X, Wu X, Chen T. Antibiotics-induced intestinal dysbacteriosis caused behavioral alternations and neuronal activation in different brain regions in mice. Mol Brain. 2021;14:49. doi: 10.1186/s13041-021-00759-w. 33676528 PMC7937204

[66] Çalışkan G, French T, Enrile Lacalle S, del Angel M, Steffen J, Heimesaat MM, Rita Dunay I, Stork O. Antibiotic-induced gut dysbiosis leads to activation of microglia and impairment of cholinergic gamma oscillations in the hippocampus. Brain Behav Immun. 2022;99:203–217. doi: 10.1016/j.bbi.2021.10.007. 34673174

[67] Li J, Pu F, Peng C, Wang Y, Zhang Y, Wu S, Shen X, Cheng R, He F. Antibiotic cocktail-induced gut microbiota depletion in different stages could cause host cognitive impairment and emotional disorders in adulthood in different manners. Neurobiol Dis. 2022;170:105757. doi: 10.1016/j.nbd.2022.105757. 35588989

[68] Neufeld KM, Kang N, Bienenstock J, Foster JA. Reduced anxiety-like behavior and central neurochemical change in germ-free mice. Neurogastroenterol Motil. 2011;23:255–e119. doi: 10.1111/j.1365-2982.2010.01620.x. 21054680

[69] Wang P, Chen Q, Yuan P, Lin S, Li R, Zhang X, Zhuo Y, Che L, Feng B, Xu S, et al. Gut microbiota involved in desulfation of sulfated progesterone metabolites: a potential regulation pathway of maternal bile acid homeostasis during pregnancy. Front Microbiol. 2022;13:1023623. doi: 10.3389/fmicb.2022.1023623. 36338075 PMC9631449

[70] Fröhlich EE, Farzi A, Mayerhofer R, Reichmann F, Jačan A, Wagner B, Zinser E, Bordag N, Magnes C, Kashofer K, et al. Cognitive impairment by antibiotic-induced gut dysbiosis: analysis of Gut microbiota-brain communication. Brain Behav Immun. 2016;56:140–155. doi: 10.1016/j.bbi.2016.02.020. 26923630 PMC5014122

[71] Hong K, Choi Y. Role of estrogen and RAS signaling in repeated implantation failure. BMB Rep. 2018;51:225–229. doi: 10.5483/BMBRep.2018.51.5.045. 29519295 PMC5988576

[72] Arevalo MA, Azcoitia I, Garcia-Segura LM. The neuroprotective actions of oestradiol and oestrogen receptors. Nat Rev Neurosci. 2015;16:17–29. doi: 10.1038/nrn3856. 25423896

[73] Vaidya SP, Li G, Chitwood RA, Li Y, Magee JC. Formation of an expanding memory representation in the hippocampus. Nat Neurosci. 2025;28:1510–1518. doi: 10.1038/s41593-025-01986-3. 40467863 PMC12229897

[74] Zhu S, Wang J, Zhang Y, He J, Kong J, Li X. The role of neuroinflammation and amyloid in cognitive impairment in an APP/PS1 transgenic mouse model of Alzheimer's disease. CNS Neurosci Ther. 2017;23:310–320. doi: 10.1111/cns.12677. 28191738 PMC6492725

[75] Xue LL, Huangfu L, Du R, Chen L, Yu C, Xiong L, Wang T. The age-specific pathological changes of β-amyloid plaques in the cortex and hippocampus of APP/PS1 transgenic AD mice. Neurol Res. 2022;44:1053–1065. doi: 10.1080/01616412.2022.2112368. 35981107

[76] Minter MR, Hinterleitner R, Meisel M, Zhang C, Leone V, Oyler-Castrillo P, Musch MW, Shen X, Jabri B, Chang EB, et al. Antibiotic-induced perturbations in microbial diversity during post-natal development alters amyloid pathology in an aged APPSWE/PS1ΔE9 murine model of Alzheimer's disease. Sci Rep. 2017;7. doi: 10.1038/s41598-017-11047-w. PMC5585265 28874832

[77] Sun B, Hu C, Li J, Yang Z, Chen L. Interaction between young fecal transplantation and perfluorobutanesulfonate endocrine disrupting toxicity in aged recipients: an estrobolome perspective. Environ Int. 2024;193:109133. doi: 10.1016/j.envint.2024.109133. 39515036

[78] Portincasa P, Bonfrate L, Vacca M, De Angelis M, Farella I, Lanza E, Khalil M, Wang DQ, Sperandio M, Di Ciaula A. Gut microbiota and short chain fatty acids: implications in glucose homeostasis. Int J Mol Sci. 2022;23:1105. doi: 10.3390/ijms23031105. 35163038 PMC8835596

[79] Wen J, Xu Q, Li J, Shen X, Zhou X, Huang J, Liu S. Sodium butyrate exerts a neuroprotective effect in rats with acute carbon monoxide poisoning by activating autophagy through the mTOR signaling pathway. Sci Rep. 2024;14(1):4610–4610. doi: 10.1038/s41598-024-55198-z. 38409245 PMC10897214

[80] Guo TT, Zhang Z, Sun Y, Zhu R, Wang F, Ma L, Jiang L, Liu H. Neuroprotective effects of sodium butyrate by restoring Gut microbiota and inhibiting TLR4 signaling in mice with MPTP-induced Parkinson's disease. Nutrients. 2023;15:930. doi: 10.3390/nu15040930. 36839287 PMC9960062

[81] Syeda T, Sanchez-Tapia M, Pinedo-Vargas L, Granados O, Cuervo-Zanatta D, Rojas-Santiago E, Díaz-Cintra S, Torres N, Perez-Cruz C, Albensi B. Bioactive food abates metabolic and synaptic alterations by modulation of Gut microbiota in a mouse model of Alzheimer's disease. J Alzheimers Dis. 2018;66:1657–1682. doi: 10.3233/JAD-180556. 30475761

