# Supplementary material for: Gut dysbiosis impacts estrogen levels in APP/PS1 transgenic female mice
Source: Gut Microbes. 2025 Dec 18;17(1):2599525. doi: 10.1080/19490976.2025.2599525 (PMC12931724; doi:10.1080/19490976.2025.2599525)
Supplement: Supplementary material — Supplementary_files_edited clean [file KGMI_A_2599525_SM2254.docx]

**Supplementary Figure S1.**


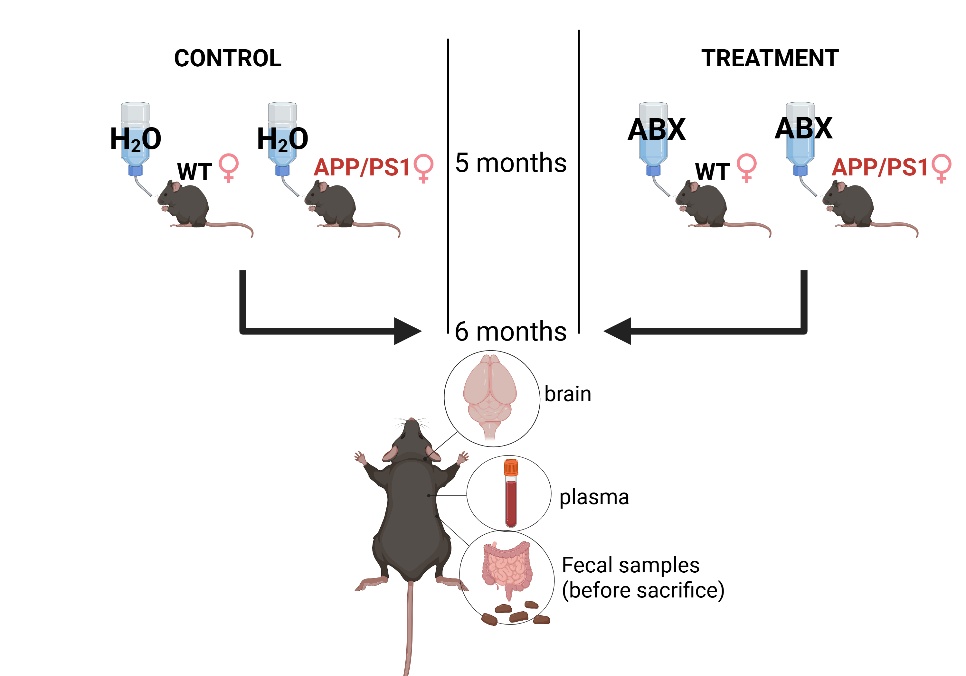


**Suppl. Figure S1) Experimental design**. Five-month-old mice were randomly assigned to two experimental groups: Control Group (vehicle), which included Wild-type (WT, n=8) and APP/PS1 (TG, n=7) mice; Treatment Group, which included antibiotic-treated WT (WT-ABX, n=7) and APP (TG-ABX, n=7) mice. ABX: A combination of antibiotics (ampicillin (1g/L), neomycin (1g/L), metronidazole (1g/L), and vancomycin (0.5 g/L)) was given in the drinking water for one month to treatment mice. C: mice were given autoclaved water following the same experimental conditions as ABX.

**Supplementary Figure S2.**


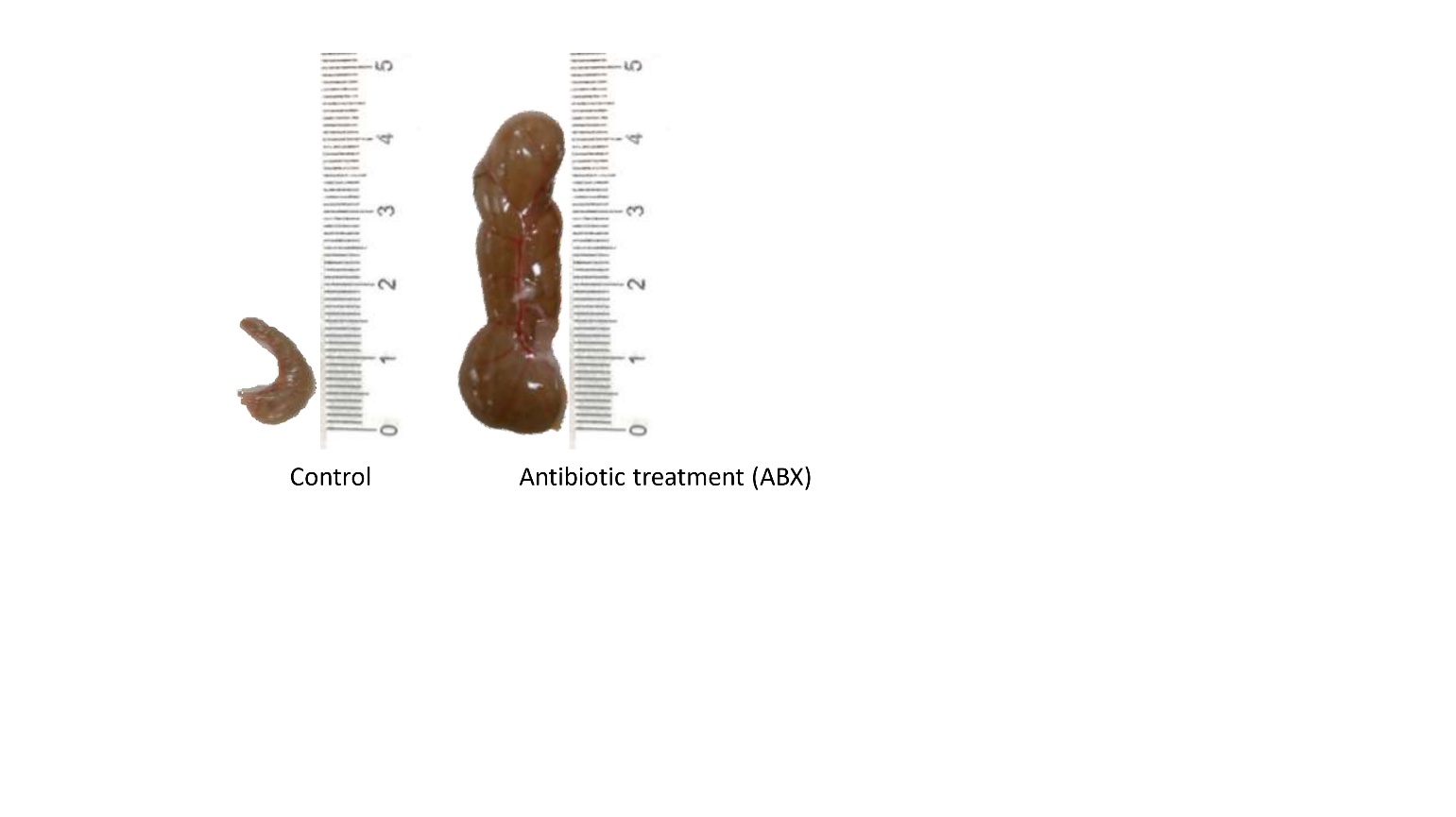


**Suppl. Figure 2S) Antibiotic administration resulted in an enlarged cecum.** Representative cecum images from control- or ABX-treated female mice.

**Supplementary Figure S3.**

**a) Total estradiol in plasma and feces**

**b) β-glucuronidase activity**

**Suppl. Figure S3) Estradiol levels and β-glucuronidase in six-month old WT and TG male mice**. **a)** Total estradiol levels were measured in plasma or fecal samples of WT and TG male mice. No significant differences were observed between groups. **b)** β-glucuronidase activity was similar between groups. Student´s t-test. For (**a**) WT n=4, TG n=6, (**b**) WT n=5, TG n=4.

**Supplementary Figure 4S**


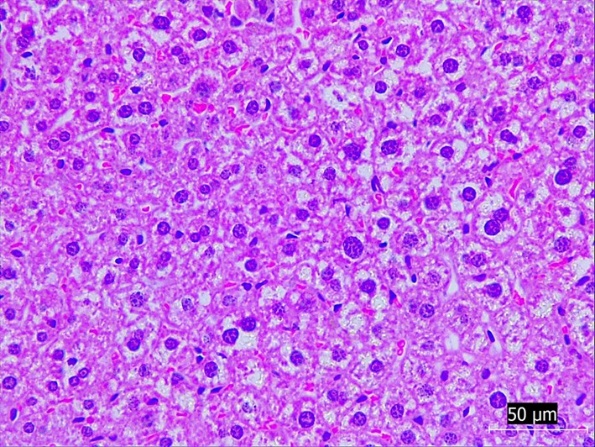

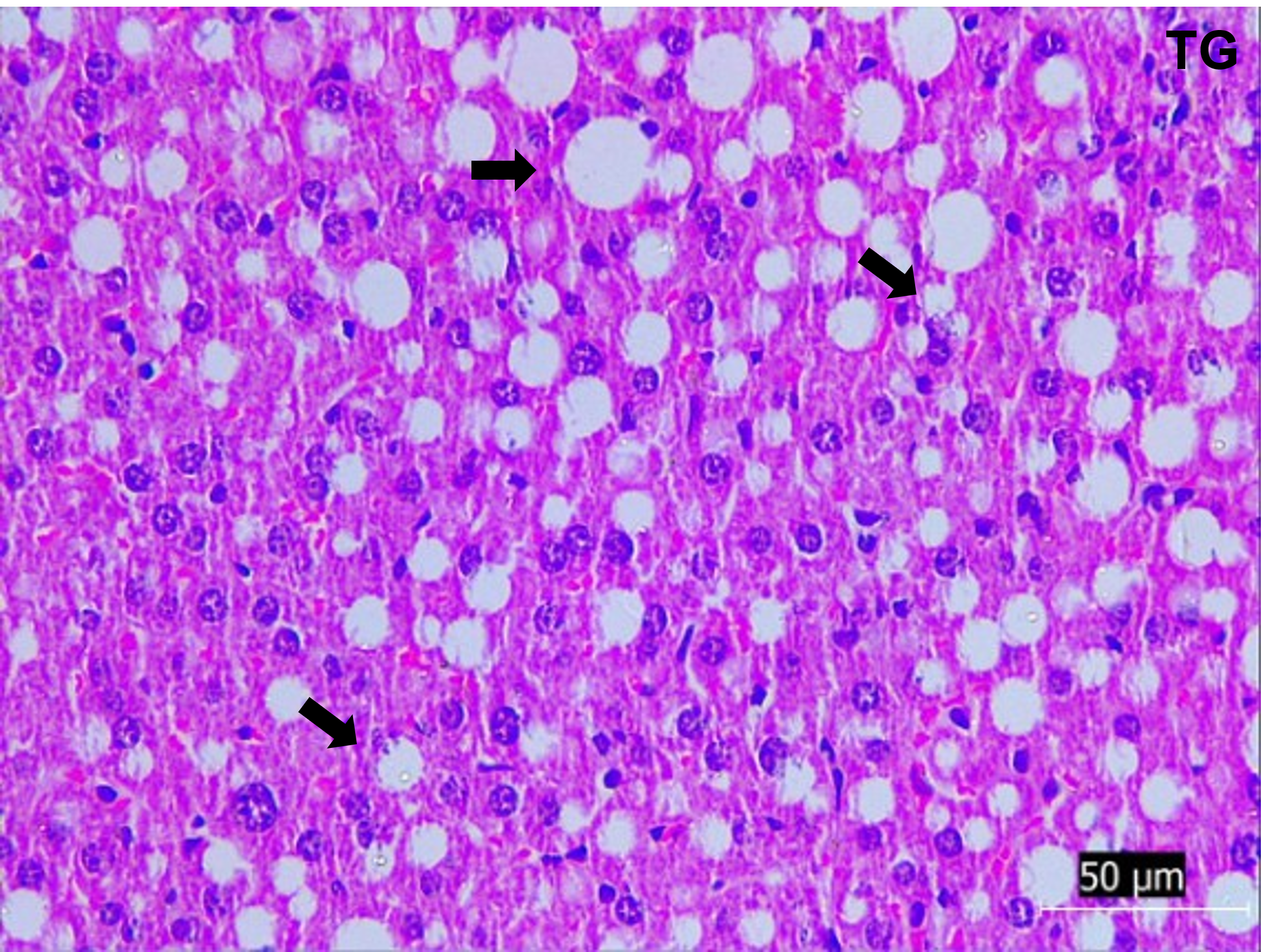


**b) Liver histology**

**a) Alanine aminotransferase activity**

**WT female mice**

**TG female mice**

**Supplementary Figure 4S. a) Alanine aminotransferase (ALT) activity in female mice.** ALT levels were determined in plasma samples from wild-type (WT) and transgenic (TG) mice from Control (vehicle) or antibiotics (ABX) treatment groups. No significant differences were detected between groups. For WT n=4, TG n=4, WT-ABX n=4, and TG-ABX n=4. Data are shown as mean ± SD bars. Two-way ANOVA with Tukey’s post-hoc correction. **b) Representative photomicrograph of female mice liver tissue stained with hematoxylin and eosin (H&E). a)** Female WT mice show normal tissue structure without visible alterations. **b)** Female TG mice show intracytoplasmic lipid vacuoles of variable size (white areas indicated by black arrows), consistent with micro- and macrovesicular hepatic steatosis (Scale bar = 50 µm). For a) WT, n=5; for b) TG, n=5.

**Supplementary tables.**

**Table S1.** Differences in the learning performance between trial 1 and trial 12.

| Trial | Group | *p*-value | Group | *p*-value |
| --- | --- | --- | --- | --- |
| 1 | WT vs. WT-ABX | <0.0001**** | WT vs. TG | <0.0001**** |
|  | TG vs. TG-ABX | 0.3547 | WT-ABX vs. TG-ABX | 0.1754 |
| 2 | WT vs. WT-ABX | 0.9994 | WT vs. TG | 0.5049 |
|  | TG vs. TG-ABX | 0.4977 | WT-ABX vs. TG-ABX | 0.9991 |
| 3 | WT vs. WT-ABX | 0.9432 | WT vs. TG | 0.1323 |
|  | TG vs. TG-ABX | 0.4078 | WT-ABX vs. TG-ABX | 0.9967 |
| 4 | WT vs. WT-ABX | 0.3122 | WT vs. TG | 0.0263* |
|  | TG vs. TG-ABX | 0.2810 | WT-ABX vs. TG-ABX | 0.9755 |
| 5 | WT vs. WT-ABX | 0.6007 | WT vs. TG | 0.0667 |
|  | TG vs. TG-ABX | 0.9913 | WT-ABX vs. TG-ABX | 0.6185 |
| 6 | WT vs. WT-ABX | 0.8024 | WT vs. TG | 0.9921 |
|  | TG vs. TG-ABX | 0.9992 | WT-ABX vs. TG-ABX | 0.8713 |
| 7 | WT vs. WT-ABX | 0.7039 | WT vs. TG | 0.1908 |
|  | TG vs. TG-ABX | 0.3568 | WT-ABX vs. TG-ABX | 0.9077 |
| 8 | WT vs. WT-ABX | 0.7134 | WT vs. TG | 0.1941 |
|  | TG vs. TG-ABX | 0.9305 | WT-ABX vs. TG-ABX | 0.9557 |
| 9 | WT vs. WT-ABX | 0.9993 | WT vs. TG | 0.5566 |
|  | TG vs. TG-ABX | 0.8687 | WT-ABX vs. TG-ABX | 0.8998 |
| 10 | WT vs. WT-ABX | 0.6995 | WT vs. TG | 0.6570 |
|  | TG vs. TG-ABX | 0.8424 | WT-ABX vs. TG-ABX | 0.8672 |
| 11 | WT vs. WT-ABX | 0.1132 | WT vs. TG | 0.0052** |
|  | TG vs. TG-ABX | 0.0293* | WT-ABX vs. TG-ABX | 0.4260 |
| 12 | WT vs. WT-ABX | 0.0043** | WT vs. TG | <0.0001**** |
|  | TG vs. TG-ABX | 0.3207 | WT-ABX vs. TG-ABX | 0.1607 |

Two-way ANOVA with Tukey’s post-hoc correction. Statistical significances are shown as **p*<0.05, ***p*<0.01, ****p*<0.001, *****p*<0.0001.

**Table S2.** Amplification conditions of the V3 region by endpoint PCR.

| Reaction components | Volume | 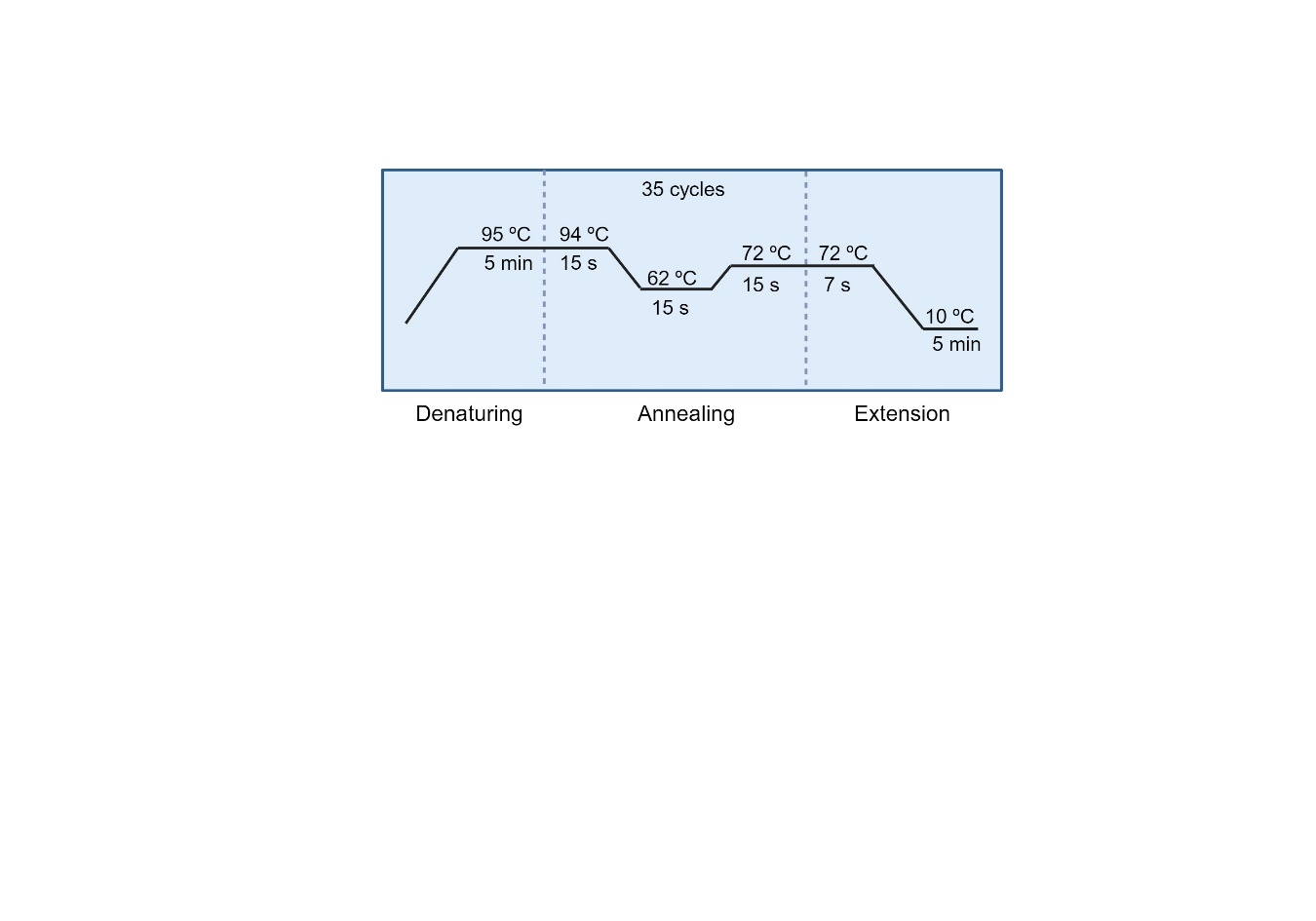 |
| --- | --- | --- |
| 5X High-Fidelity Buffer | 4 μL |  |
| 200 μM dNTP mix | 0.4 μL |  |
| 0.5 μM Reverse primer | 1 μL |  |
| 0.5 μM Forward primer | 1 μL |  |
| 2 U/ μL Phusion DNA polymerase | 0.2 μL |  |
| 100 % DMSO | 0.6 μL |  |
| DNA genome | 1 ng/μL |  |

**Table S3.** Relative abundance at phylum level in the experimental groups

| Phylum | Group | % | SD | %CV | Range |  | *p*-value |
| --- | --- | --- | --- | --- | --- | --- | --- |
| Actinobacteriota | WT | 27.45 | 11.11 | 55.31 | 7.29 – 39.14 | WT *vs.* WT-ABX | 0.0047 ** |
|  | WT-ABX | 10.25 | 8.93 | 143.1 | 0.23 – 20.42 | WT *vs.* TG | 0.7654 |
|  | TG | 18.94 | 5.14 | 24.71 | 13.30 – 24.97 | TG *vs.* TG-ABX | 0.0026 ** |
|  | TG-ABX | 0.71 | 0.23 | 16.88 | 1.16 – 1.64 | WT-ABX *vs.* TG-ABX | 0.2925 |
| Bacteroidota | WT | 1.71 | 0.18 | 32.20 | 0.36 – 0.91 | WT *vs.* WT-ABX | 0.0379 * |
|  | WT-ABX | 0 | 0 | 0 | 0 | WT *vs.* TG | 0.3385 |
|  | TG | 5.98 | 1.61 | 25.47 | 4.15 – 7.88 | TG *vs.* TG-ABX | 0.0028 ** |
|  | TG-ABX | 0 | 0 | 0 | 0 | WT-ABX *vs.* TG-ABX | 0.9886 |
| Cyanobacteria | WT | 0 | 0 | 0 | 0 | WT *vs.* WT-ABX | 0.6717 |
|  | WT-ABX | 4.27 | 3.63 | 71.16 | 0.05 – 8.51 | WT *vs.* TG | 0.9989 |
|  | TG | 0 | 0 | 0 | 0 | TG *vs.* TG-ABX | <0.0001*** |
|  | TG-ABX | 37.43 | 15.66 | 32.27 | 25.98 – 61.86 | WT-ABX *vs.* TG-ABX | 0.0004 |
| Firmicutes_A | WT | 4.97 | 2.33 | 56.02 | 2.24 – 7.62 | WT *vs.* WT-ABX | 0.2625 |
|  | WT-ABX | 16.27 | 11.81 | 89.52 | 0 – 32-42 | WT *vs.* TG | 0.5220 |
|  | TG | 20.51 | 9.45 | 70.91 | 5.97 – 27.03 | TG *vs.* TG-ABX | 0.1068 |
|  | TG-ABX | 0.92 | 0.25 | 21.70 | 1.00 – 1.52 | WT-ABX *vs.* TG-ABX | 0.0516 |
| Firmicutes_D | WT | 65.35 | 24.70 | 39.64 | 25.03 – 93.18 | WT *vs.* WT-ABX | 0.0296 * |
|  | WT-ABX | 37.07 | 17.87 | 33.91 | 32.90 – 73.87 | WT *vs.* TG | 0.0014 ** |
|  | TG | 51.22 | 23.37 | 56.88 | 18.15 – 67.51 | TG *vs.* TG-ABX | 0.2390 |
|  | TG-ABX | 45.69 | 23.47 | 42.64 | 24.20 – 75.49 | WT-ABX *vs.* TG-ABX | 0.9966 |
| Proteobacteriota | WT | 0 | 0 | 0 | 0 | WT *vs.* WT-ABX | 0.0019 ** |
|  | WT-ABX | 31.79 | 24.04 | 10.75 | 0 – 63.36 | WT *vs.* TG | 0.9308 |
|  | TG | 0 | 0 | 0 | 0 | TG *vs.* TG-ABX | 0.1072 |
|  | TG-ABX | 13.87 | 1.82 | 8.81 | 18.48 – 22.92 | WT-ABX *vs.* TG-ABX | 0.0843 |
| Verrucomicrobiota | WT | 0 | 0 | 0 | 0 | WT *vs.* WT-ABX | > 0.9999 |
|  | WT-ABX | 0 | 0 | 0 | 0 | WT *vs.* TG | <0.0001**** |
|  | TG | 3.04 | 0.74 | 20.62 | 2.50 – 4.01 | TG *vs.* TG-ABX | <0.0001**** |
|  | TG-ABX | 0 | 0 | 0 | 0 | WT-ABX *vs.* TG-ABX | > 0.9999 |

Two-way ANOVA with Tukey’s post-hoc correction. Statistical significances are shown as **p*<0.05,***p*<0.01, ****p*<0.001, *****p*<0.0001.

**Table S4.** Relative abundance at genus level in the experimental groups

| Genus | Group | % | SD | %CV | Range |  | *p*-value |
| --- | --- | --- | --- | --- | --- | --- | --- |
| *Akkermansia* | WT | 0 | 0 | 0 | 0 | WT *vs.* WT-ABX | >0.9999 |
|  | WT-ABX | 0 | 0 | 0 | 0 | WT *vs.* TG | <0.0001**** |
|  | TG | 3.67 | 0.80 | 21.74 | 2-76 – 4.85 | TG *vs.* TG-ABX | <0.0001**** |
|  | TG-ABX | 0 | 0 | 0 | 0 | WT-ABX *vs.* TG-ABX | >0.9999 |
| *Anaerostipes* | WT | 0 | 0 | 0 | 0 | WT *vs.* WT-ABX | >0.9999 |
|  | WT-ABX | 0 | 0 | 0 | 0 | WT *vs.* TG | <0.0001**** |
|  | TG | 1.81 | 0.68 | 37.78 | 1.03 – 2.53 | TG *vs.* TG-ABX | <0.0001**** |
|  | TG-ABX | 0 | 0 | 0 | 0 | WT-ABX *vs.* TG-ABX | >0.9999 |
| *Bifidobacterium_388775* | WT | 0.23 | 0.06 | 27.84 | 0.17 – 0.33 | WT *vs.* WT-ABX | <0.0001*** |
|  | WT-ABX | 7.64 | 2.05 | 26.87 | 4.59 – 9.04 | WT *vs.* TG | 0.9992 |
|  | TG | 0 | 0 | 0 | 0 | TG *vs.* TG-ABX | >0.9999 |
|  | TG-ABX | 0 | 0 | 0 | 0 | WT-ABX *vs.* TG-ABX | <0.0001**** |
| *CAG-314* | WT | 0 | 0 | 0 | 0 | WT *vs.* WT-ABX | <0.0001**** |
|  | WT-ABX | 29.38 | 2.69 | 9.15 | 25.84 – 32.36 | WT *vs.* TG | 0.9633 |
|  | TG | 0 | 0 | 0 | 0 | TG *vs.* TG-ABX | >0.9999 |
|  | TG-ABX | 0 | 0 | 0 | 0 | WT-ABX *vs.* TG-ABX | <0.0001**** |
| *CAG-485* | WT | 0 | 0 | 0 | 0 | WT *vs.* WT-ABX | <0.0001**** |
|  | WT-ABX | 6.64 | 1.60 | 24.16 | 4.58 – 8.71 | WT *vs.* TG | 0.0001*** |
|  | TG | 3.76 | 1.37 | 36.54 | 2.07 – 5.01 | TG *vs.* TG-ABX | 0.0002*** |
|  | TG-ABX | 0 | 0 | 0 | 0 | WT-ABX *vs.* TG-ABX | <0.0001**** |
| *Cupriavidus* | WT | 0 | 0 | 0 | 0 | WT *vs.* WT-ABX | <0.0001**** |
|  | WT-ABX | 29.90 | 9.60 | 32.11 | 14.09 – 42.87 | WT *vs.* TG | 0.9996 |
|  | TG | 0 | 0 | 0 | 0 | TG *vs.* TG-ABX | 0.0061** |
|  | TG-ABX | 16.16 | 7.83 | 48.43 | 2.88 – 22.92 | WT-ABX *vs.* TG-ABX | 0.0136* |
| *Dubosiella* | WT | 21.89 | 7.73 | 35.29 | 7.20 – 32.02 | WT *vs.* WT-ABX | <0.0001**** |
|  | WT-ABX | 3.22 | 0.94 | 29.22 | 1.85 – 4.53 | WT *vs.* TG | 0.0005* |
|  | TG | 6.94 | 2.56 | 36.80 | 2.67 – 9.10 | TG *vs.* TG-ABX | 0.1557 |
|  | TG-ABX | 0.71 | 0.30 | 42.87 | 0.18 – 0.94 | WT-ABX *vs.* TG-ABX | 0.8092 |
| *Enterococcus_H_360604* | WT | 0 | 0 | 0 | 0 | WT *vs.* WT-ABX | >0.9999 |
|  | WT-ABX | 0 | 0 | 0 | 0 | WT *vs.* TG | >0.9999 |
|  | TG | 0 | 0 | 0 | 0 | TG *vs.* TG-ABX | <0.0001**** |
|  | TG-ABX | 62.29 | 14.35 | 23.04 | 40.65 – 75.01 | WT-ABX *vs.* TG-ABX | <0.0001**** |
| *Escherichia_ 710834* | WT | 0 | 0 | 0 | 0 | WT *vs.* WT-ABX | <0.0001**** |
|  | WT-ABX | 33.74 | 9.65 | 28.60 | 22 – 49.27 | WT *vs.* TG | 0.9983 |
|  | TG | 0 | 0 | 0 | 0 | TG *vs.* TG-ABX | >0.9999 |
|  | TG-ABX | 0 | 0 | 0 | 0 | WT-ABX *vs.* TG-ABX | <0.0001**** |
| *Faecalibaculum* | WT | 0 | 0 | 0 | 0 | WT *vs.* WT-ABX | <0.0001**** |
|  | WT-ABX | 14.64 | 0.74 | 5.08 | 13.21 – 15.18 | WT *vs.* TG | 0.0027** |
|  | TG | 1.18 | 0.25 | 20.92 | 1.00 – 1.52 | TG *vs.* TG-ABX | 0.0036** |
|  | TG-ABX | 0 | 0 | 0 | 0 | WT-ABX *vs.* TG-ABX | <0.0001**** |
| *Ileibacterium* | WT | 4.99 | 4.26 | 85.21 | 1.022 – 11.30 | WT *vs.* WT-ABX | 0.0131* |
|  | WT-ABX | 0.25 | 0.05 | 18.61 | 0.20 – 0.30 | WT *vs.* TG | 0.1575 |
|  | TG | 3.33 | 2.82 | 84.70 | 1.28 – 7.58 | TG *vs.* TG-ABX | 0.1593 |
|  | TG-ABX | 0 | 0 | 0 | 0 | WT-ABX *vs.* TG-ABX | 0.8223 |

| Genus | Group | % | SD | %CV | Range |  | *p*-value |
| --- | --- | --- | --- | --- | --- | --- | --- |
| *Kineothrix* | WT | 0 | 0 | 0 | 0 | WT *vs.* WT-ABX | >0.9999 |
|  | WT-ABX | 0 | 0 | 0 | 0 | WT *vs.* TG | <0.0001**** |
|  | TG | 3.57 | 0.16 | 4.54 | 3.30 – 3.73 | TG *vs.* TG-ABX | <0.0001**** |
|  | TG-ABX | 0 | 0 | 0 | 0 | WT-ABX *vs.* TG-ABX | >0.9999 |
| *Lactobacillus* | WT | 32.69 | 17.00 | 51.99 | 15.42 – 66.45 | WT *vs.* WT-ABX | 0.0057** |
|  | WT-ABX | 8.16 | 0.67 | 8.18 | 7.12 – 8.85 | WT *vs.* TG | 0.0047** |
|  | TG | 11.45 | 2.70 | 23.60 | 8.48 – 15.52 | TG *vs.* TG-ABX | 0.0023** |
|  | TG-ABX | 35.65 | 9.70 | 26.45 | 30.07 – 52.28 | WT-ABX *vs.* TG-ABX | 0.0041** |
| *Lactococcus_A_346120* | WT | 0 | 0 | 0 | 0 | WT *vs.* WT-ABX | <0.0001**** |
|  | WT-ABX | 1.81 | 0.69 | 38.18 | 1.28 – 3.19 | WT *vs.* TG | 0.9998 |
|  | TG | 0 | 0 | 0 | 0 | TG *vs.* TG-ABX | <0.0001**** |
|  | TG-ABX | 2.87 | 0.53 | 18.50 | 1.93 – 3.22 | WT-ABX *vs.* TG-ABX | 0.0111* |
| *Ligilactobacillus* | WT | 1.83 | 0.88 | 48.31 | 1.08 – 3.30 | WT *vs.* WT-ABX | 0.0232* |
|  | WT-ABX | 0 | 0 | 0 | 0 | WT *vs.* TG | 0.0009*** |
|  | TG | 4.98 | 1.76 | 35.37 | 2.02 – 6.15 | TG *vs.* TG-ABX | <0.0001**** |
|  | TG-ABX | 0.34 | 0.16 | 48.75 | 0.13 – 0.51 | WT-ABX *vs.* TG-ABX | 0.8797 |
| *Limosilactobacillus* | WT | 9.80 | 4.43 | 45.25 | 3.33 – 18.34 | WT *vs.* WT-ABX | 0.0006*** |
|  | WT-ABX | 1.16 | 0.11 | 9.24 | 1.00 – 1.26 | WT *vs.* TG | 0.0143* |
|  | TG | 4.65 | 1.69 | 36.42 | 2.92 – 7.32 | TG *vs.* TG-ABX | 0.2368 |
|  | TG-ABX | 7.64 | 0.73 | 9.59 | 6.40 – 8.31 | WT-ABX *vs.* TG-ABX | 0.0161* |
| *Parabacteroides_B_862066* | WT | 0 | 0 | 0 | 0 | WT *vs.* WT-ABX | <0.0001**** |
|  | WT-ABX | 27.77 | 2.49 | 8.98 | 24.69 – 30.77 | WT *vs.* TG | > 0.9999 |
|  | TG | 0 | 0 | 0 | 0 | TG *vs.* TG-ABX | > 0.9999 |
|  | TG-ABX | 0 | 0 | 0 | 0 | WT-ABX *vs.* TG-ABX | <0.0001**** |
| *Schaedlerella* | WT | 0 | 0 | 0 | 0 | WT *vs.* WT-ABX | <0.0001**** |
|  | WT-ABX | 4.25 | 1.58 | 37.23 | 1.37 – 5.69 | WT *vs.* TG | 0.9998 |
|  | TG | 0 | 0 | 0 | 0 | TG *vs.* TG-ABX | > 0.9999 |
|  | TG-ABX | 0 | 0 | 0 | 0 | WT-ABX *vs.* TG-ABX | <0.0001**** |
| *SIO2CI* | WT | 0 | 0 | 0 | 0 | WT *vs.* WT-ABX | 0.2409 |
|  | WT-ABX | 8.33 | 3.46 | 41.47 | 5.18 – 15.02 | WT *vs.* TG | > 0.9999 |
|  | TG | 0 | 0 | 0 | 0 | TG *vs.* TG-ABX | <0.0001**** |
|  | TG-ABX | 34.90 | 15.79 | 45.26 | 24.66 – 61.86 | WT-ABX *vs.* TG-ABX | 0.0003 |
| *Streptococcus* | WT | 0 | 0 | 0 | 0 | WT *vs.* WT-ABX | > 0.9999 |
|  | WT-ABX | 0 | 0 | 0 | 0 | WT *vs.* TG | > 0.9999 |
|  | TG | 0 | 0 | 0 | 0 | TG *vs.* TG-ABX | <0.0001**** |
|  | TG-ABX | 3.31 | 1.06 | 31.93 | 1.59 – 4.15 | WT-ABX *vs.* TG-ABX | <0.0001**** |
| *Turicibacter* | WT | 17.95 | 5.83 | 32.44 | 10.73 – 25.29 | WT *vs.* WT-ABX | 0.0002*** |
|  | WT-ABX | 7.07 | 2.19 | 30.93 | 4.11 – 10.23 | WT *vs.* TG | 0.7783 |
|  | TG | 16.72 | 1.78 | 10.63 | 14.25 – 18.98 | TG *vs.* TG-ABX | <0.0001**** |
|  | TG-ABX | 0 | 0 | 0 | 0 | WT-ABX *vs.* TG-ABX | 0.0074** |
| *UMGS1994* | WT | 0 | 0 | 0 | 0 | WT *vs.* WT-ABX | > 0.9999 |
|  | WT-ABX | 0 | 0 | 0 | 0 | WT *vs.* TG | <0.0001**** |
|  | TG | 2.75 | 0.93 | 33.84 | 1.77 – 3.95 | TG *vs.* TG-ABX | <0.0001**** |
|  | TG-ABX | 0 | 0 | 0 | 0 | WT-ABX *vs.* TG-ABX | > 0.9999 |

Two-way ANOVA with Tukey’s post-hoc correction. Statistical significances are shown as **p*<0.05, ***p*<0.01, ****p*<0.001, *****p*<0.0001.

**Table S5.** Alpha diversity indices in the experimental groups.

| Indices | Group | % | SD | %CV | Range |  | *p*-value |
| --- | --- | --- | --- | --- | --- | --- | --- |
| CHAO1 | WT | 54.43 | 13.87 | 25.48 | 38.00 – 82.00 | WT *vs.* WT-ABX | 0.0121 * |
|  | WT-ABX | 24.00 | 14.02 | 58.41 | 12.00 – 47.00 | WT *vs.* TG | 0.9980 |
|  | TG | 56.20 | 12.89 | 22.94 | 41.00 – 74.00 | TG *vs.* TG-ABX | 0.0084** |
|  | TG-ABX | 20.25 | 6.80 | 33.58 | 13.00 – 27.00 | WT-ABX *vs.* TG-ABX | 0.9848 |
| ACE | WT | 54.43 | 13.87 | 25.48 | 38.00 _ 82.00 | WT *vs.* WT-ABX | 0.0121 * |
|  | WT-ABX | 24.00 | 14.02 | 58.41 | 12.00 – 47.00 | WT *vs.* TG | 0.9980 |
|  | TG | 56.20 | 12.89 | 22.94 | 41.00 – 74.00 | TG *vs.* TG-ABX | 0.0084** |
|  | TG-ABX | 20.25 | 6.80 | 33.58 | 13.00 – 27.00 | WT-ABX *vs.* TG-ABX | 0.9848 |
| Shannon | WT | 2.40 | 0.50 | 21.03 | 1.47 – 3.00 | WT *vs.* WT-ABX | 0.5251 |
|  | WT-ABX | 1.81 | 0.57 | 31.80 | 1.28 – 2.44 | WT *vs.* TG | 0.0858 |
|  | TG | 3.12 | 0.08 | 2.70 | 3.04 – 3.21 | TG *vs.* TG-ABX | 0.0030** |
|  | TG-ABX | 1.54 | 0.69 | 44.94 | 0.68 – 2.40 | WT-ABX *vs.* TG-ABX | 0.8518 |
| Simpson | WT | 0.85 | 0.05 | 6.23 | 0.79 – 0.92 | WT *vs.* WT-ABX | 0.6745 |
|  | WT-ABX | 0.75 | 0.10 | 13.78 | 0.64 – 0.86 | WT *vs.* TG | 0.8121 |
|  | TG | 0.90 | 0.02 | 2.18 | 0.87 – 0.93 | TG *vs.* TG-ABX | 0.0066** |
|  | TG-ABX | 0.61 | 0.17 | 27.11 | 0.39 – 0.74 | WT-ABX *vs.* TG-ABX | 0.2314 |
| Fisher | WT | 7.47 | 2.01 | 26.91 | 5.32 – 11.43 | WT *vs.* WT-ABX | 0.0281 * |
|  | WT-ABX | 3.21 | 1.99 | 62.11 | 1.44 – 6.14 | WT *vs.* TG | 0.6480 |
|  | TG | 9.18 | 2.56 | 27.89 | 5.40 – 12.09 | TG *vs.* TG-ABX | 0.0021** |
|  | TG-ABX | 2.20 | 1.09 | 49.49 | 1.50 – 3.82 | WT-ABX *vs.* TG-ABX | 0.8722 |

Two-way ANOVA with Tukey’s post-hoc correction. Statistical significances are shown as **p*<0.05, ***p*<0.01, ****p*<0.001, *****p*<0.0001.
